# Hypodermal responses to protein synthesis inhibition induce systemic developmental arrest and AMPK-dependent survival in *Caenorhabditis elegans*

**DOI:** 10.1371/journal.pgen.1007520

**Published:** 2018-07-18

**Authors:** Hans M. Dalton, Sean P. Curran

**Affiliations:** 1 Leonard Davis School of Gerontology, University of Southern California, Los Angeles, California, United States of America; 2 Dornsife College of Letters, Arts, and Sciences, University of Southern California, Los Angeles, California, United States of America; 3 Norris Comprehensive Cancer Center, Keck School of Medicine, University of Southern California, Los Angeles, California, United States of America; Princeton, UNITED STATES

## Abstract

Across organisms, manipulation of biosynthetic capacity arrests development early in life, but can increase health- and lifespan post-developmentally. Here we demonstrate that this developmental arrest is not sickness but rather a regulated survival program responding to reduced cellular performance. We inhibited protein synthesis by reducing ribosome biogenesis (*rps-11*/*RPS11* RNAi), translation initiation (*ifg-1*/*EIF3G* mutation and *egl-45*/*EIF3A* RNAi), or ribosome progression (cycloheximide treatment), all of which result in a specific arrest at larval stage 2 of *C*. *elegans* development. This quiescent state can last for weeks—beyond the normal *C*. *elegans* adult lifespan—and is reversible, as animals can resume reproduction and live a normal lifespan once released from the source of protein synthesis inhibition. The arrest state affords resistance to thermal, oxidative, and heavy metal stress exposure. In addition to cell-autonomous responses, reducing biosynthetic capacity only in the hypodermis was sufficient to drive organism-level developmental arrest and stress resistance phenotypes. Among the cell non-autonomous responses to protein synthesis inhibition is reduced pharyngeal pumping that is dependent upon AMPK-mediated signaling. The reduced pharyngeal pumping in response to protein synthesis inhibition is recapitulated by exposure to microbes that generate protein synthesis-inhibiting xenobiotics, which may mechanistically reduce ingestion of pathogen and toxin. These data define the existence of a transient arrest-survival state in response to protein synthesis inhibition and provide an evolutionary foundation for the conserved enhancement of healthy aging observed in post-developmental animals with reduced biosynthetic capacity.

## Author summary

Protein synthesis is an essential cellular process, but post-developmental reduction of protein synthesis across multiple species leads to improved health- and lifespan. To better understand the physiological responses to impaired protein synthesis, we characterize a novel developmental arrest state that occurs when reducing protein synthesis during *C*. *elegans* development. Arrested animals have multiple survival-promoting phenotypes that are all dependent on the cellular energy sensor, AMP kinase. This survival response acts through the hypodermis and causes a reduction in pharyngeal pumping, indicating that the animal is responding to a perceived external threat, even in adults. Furthermore, exposing animals to pathogens, or xenobiotics they produce, can recapitulate these phenotypes, providing a potential evolutionary explanation for how a beneficial response in adults could evolve through the inhibition of an essential biological process such as protein synthesis.

## Introduction

The differing phenotypes stemming from the loss of essential cellular functions, such as protein synthesis, are specific to the time in life (development or adulthood) when the deficit occurs. Under such deficits, arresting development is an established strategy at the disposal of animals to ensure future reproductive success.

During its four larval stages, the nematode *C*. *elegans* has several possible arrested states that trigger in response to different stressors, including dauer [1, 2], starvation-induced arrest [3], and adult reproductive diapause [4, 5], among others. Dauer diapause occurs under lack of food, high temperature, or high population density, inducing an alternative larval stage 3 [2]; this dauer state carries both metabolic and behavioral changes, including increased stress resistance [6, 7]. This stress resistant and pre-reproductive arrest state is thought to have evolved to allow the worm to conserve its resources, and it affords protection from the environment until a more favorable environment is encountered. Starvation-induced arrest can occur at larval stage 1 (L1), induced from starvation occurring immediately after hatching, and this state similarly results in stress resistance[3]. Two other arrest states are adult reproductive diapause, which is induced by L4 starvation and results in an early-adult arrest state capable of surviving long periods of nutrient deprivation with the ability to later resume reproduction, and impaired mitochondria arrest, induced by deficiency in mitochondrial respiration and resulting in L3 arrest [4, 8]; however, these two states have not yet been directly shown to have stress resistance phenotypes. These examples suggest the existence of cellular programs that function as checkpoints throughout development that stall reproduction to promote fitness [9].

Intriguingly, the same triggers that induce these genetically regulated arrest states during development, when initiated post-developmentally, lead to increased life and healthspan (e.g. *daf-2*/Insulin IGFI signaling mutants [10–12], mitochondrial deficiency [13, 14]). Moreover, the loss of essential cellular functions was shown to alter animal behavior [15], presumably to avoid further exposure to the environment causal for the perceived loss of cellular homeostasis [16–18].

Protein synthesis inhibition is another trigger of developmental arrest early in life and increased lifespan in adults [16, 19–21], although the underlying mechanisms are not well understood. Similar to inhibiting the insulin-signaling pathway in adults, inhibiting protein synthesis provides several resistances from stress—starvation, thermal, and oxidative [20, 22]. Activation of the energy sensor AMP-activated protein kinase (AMPK) is linked to a reduction in protein synthesis [23–25], and AMPK can be activated by reducing growth via starvation in *C*. *elegans* [26] or via inhibiting S6 kinase in isolated mouse cells [27, 28]; this activation includes increased lifespan that is dependent on activation of AMPK in *C*. *elegans* [28].

Here we provide new characterization of a *C*. *elegans* survival arrest state brought on by reducing protein synthesis, which confers stress resistance and is reversible. Enacting protein synthesis inhibition in the hypodermis alone was partially sufficient for both the arrest and stress resistance phenotypes. Arrested animals had very high expression of a metallothionein and were found to have higher levels of calcium, which may be linked to an observed reduction in pharyngeal pumping. All of these survival phenotypes, save the arrest, were dependent on functional AMPK. Finally, these phenotypes could be recapitulated from exposure to xenobiotics, implying a potential evolutionary context for this fitness-promoting arrest state.

## Results

### Protein synthesis inhibition induces a stress resistant developmental arrest state

**Fig 1:**
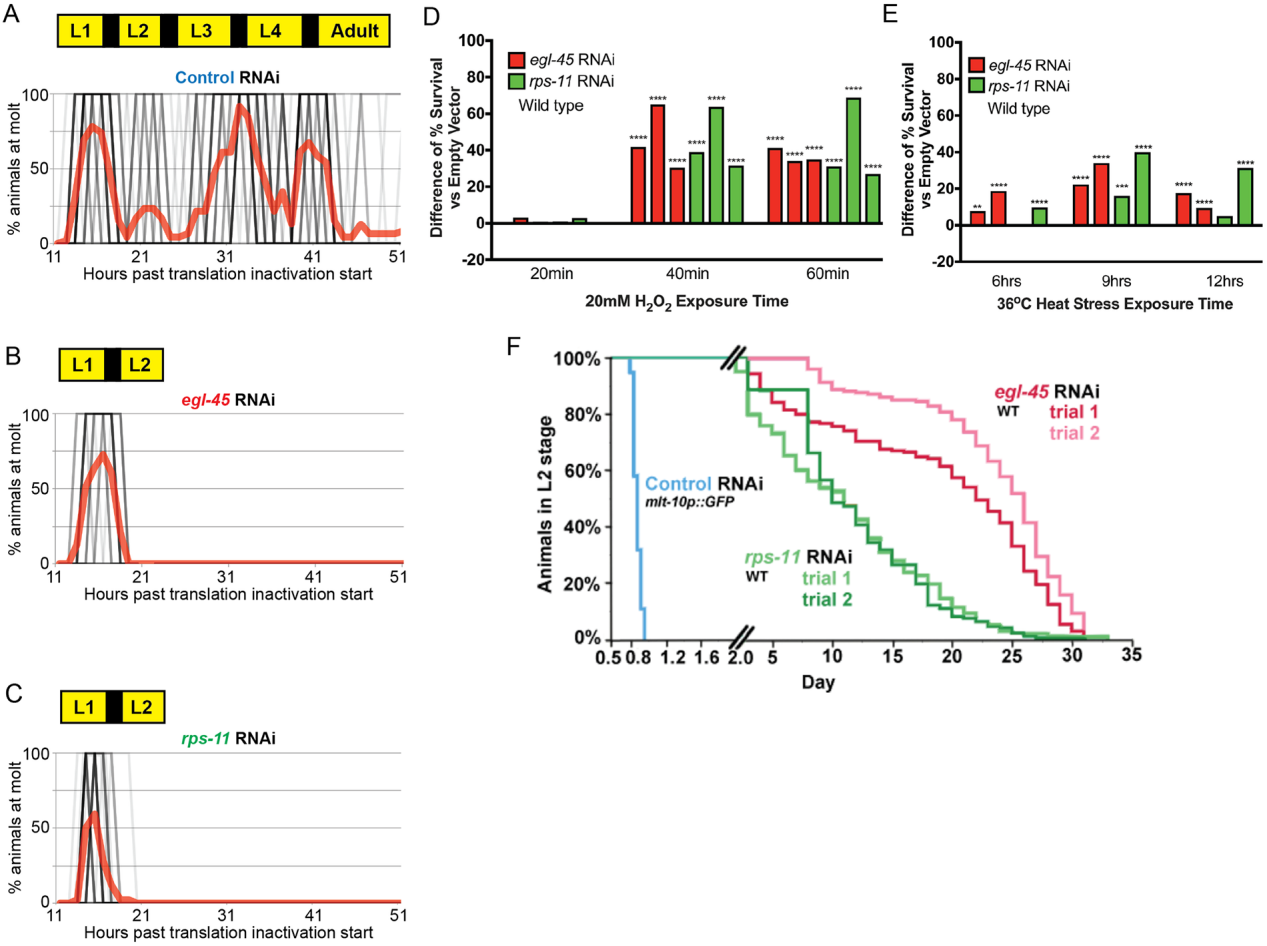
Protein synthesis inhibition promotes a developmental arrest and survival state. *A*-*C*. *C*. *elegans* normally transit temporally through four larval stages; each is stage separated by a molt (*A*), while protein synthesis inhibition induced by *egl-45* RNAi (*B*) or *rps-11* RNAi (*C*) results in arrest at larval stage 2 (L2); each black line represents a single worm (darker shades indicating more animals) and the red line represents % molting worms in the population. Note that starting from synchronized L1s, Control RNAi animals go through four molts (represented by each "peak" of red) to become Adults, while *egl-45* and *rps-11* RNAi treated animals cease development after the first molt as L2 stage larva (N = 33–46 from 2 biological replicates). *D*-*E*. Protein synthesis inhibition-induced arrested L2 larvae are resistant to oxidative (*D*) and thermal (*E*) stress as compared to control RNAi treated L2 stage animals (the bars in each graph represent a unique set of biological replicates [S1 Table] relative to its own independent control cohort, N = 126–251 from 2–3 biological replicates). (*F*). Wild type L2 stage lasts ~8hrs (panel A), while arrested animals survive weeks in the L2 stage; WT animals exit L2 stage by proceeding to L3 stage and normal development, while arrested animals "exit" L2 stage via death (N = 180–227 from 2–3 biological replicates). * p< 0.025, ** p<0.005, *** p<0.0005, **** p<0.00005 (*D*-*E*: Fisher's exact test). See also S1 Table.

To elucidate the possible connection between the developmental arrest and longevity-promoting effects of protein synthesis inhibition [16, 19–21, 29], we first defined the nature of the developmental arrest in *C*. *elegans*. We analyzed the effects of protein synthesis inhibition by targeting distinct and conserved aspects of the protein biosynthesis machinery (S1A Fig). We measured the synthesis of two GFP reporters; a heat shock inducible promoter (S1B Fig) and a *mlt-10p* driven construct (S1C Fig) that is only expressed between developmental molts as a surrogate assessment for general protein biosynthesis [30]. Because GFP from these reporters is limited to temporally distinct periods, we can robustly measure differences in GFP levels between protein synthesis inhibition conditions. We targeted the translation initiation factor, *egl-45*/*EIF3A*, or the small ribosomal protein, *rps-11*/*RPS11*, by RNA interference (RNAi), so that we could control the strength and duration of inhibition, thereby avoiding the constitutive arrest that can occur when protein synthesis is inhibited by genetic mutation [31]. While there are many genes involved in protein synthesis that can induce arrest when inhibited [16, 19, 20], *egl-45* and *rps-11* were selected as RNAi of these genes results in a fully penetrant larval arrest phenotype (S1D Fig). There is a threshold effect to this arrest, as diluting the RNAi to 10% of total food allowed more escaping animals (S1E Fig), while still impairing development. In all RNAi conditions tested at 100% of total food, we observed a potent developmental arrest that could persist beyond 10 days (S1D Fig). To define the developmental arrest state more precisely, we made use of the molting reporter (*mlt-10p*::*gfp-pest*) that marks each of the four developmental molts in *C*. *elegans* [32]. This revealed a potent arrest after the first molt at larval stage 2 (L2) (Fig 1A–1C). In addition, these animals are morphologically different than other arrest states like dauer and L1 arrested animals (S1F Fig) and are smaller in length than wild type L2s; unlike arrested L2d animals [33] (S1G Fig). Together, these data support the existence of a potent developmental arrest point in response to diminished biosynthetic capacity.

To address the hypothesis that the induced developmental arrest in response to protein synthesis inhibition is beneficial, we challenged L2 arrested animals and non-arrested L2 control animals to oxidative (20mM H_2_O_2_, Fig 1D and S2A Fig) or thermal (36°C, Fig 1E and S2B Fig) stress and found the arrested animals were more resistant to all tested environmental insults. Animals that remained in the arrested state for longer periods of time (2 or 10 days) were markedly more protected against oxidative stress and extended exposures to thermal stress (S2C–S2F Fig). Thus, the durability of the response and the capacity to further enhance resistance to perceived deficiencies is enhanced so long as it is needed. Collectively, these data show that loss of protein biosynthetic capacity during development does not induce a decrepit state, but rather a beneficial health-promoting state of impeded development.

The amplification of stress resistance that correlated with time in the arrested state predicted that arrested animals could persist in the L2 stage for much longer than wild type animals. Given this, we examined the lifespan of animals in the arrested state and discovered that *egl-45* RNAi and *rps-11* RNAi animals had a mean survival in the arrested state of 24 and 12 days, respectively (Fig 1F), compared to a normal eight hour L2 stage (Fig 1A). As such, the developmental arrest resulting from reduction of protein biosynthetic capacity results in health-promoting state of extended diapause.

One hypothesis is that pausing development in the L2 stage alone confers survival benefits. To test this, we screened all annotated RNAi clones that induce early and fully penetrant L2 arrest (S2G and S2H Fig) and measured their ability to resist the same exposure to stress. Despite sharing an L2 arrest phenotype, none of these RNAi treatments resulted in the same decrease in protein synthesis (S2I Fig) or afforded increased survival during stress (S2J and S2K Fig). As such, arrest at the L2 stage does not require a loss in biosynthetic capacity and is not inherently stress resistance-promoting. In addition, the phenotypes observed are not tied to RNAi responses, as *ifg-1(ok1211)* mutant animals that arrest at the L2 state [31] are more resistant to oxidative stress as compared to wild type controls (S2L Fig). We also tested the long-term survival of *acn-1*, *let-767*, and *pan-1*; while only *acn-1* maintained long-term L2 arrest (S2H Fig), the survival of *acn-1* RNAi treated animals was significantly shorter than *rps-11* and *egl-45* RNAi treated animals (S2M Fig).

Finally, we tested the necessity of *daf-16*/*FOXO*, a transcription factor that is required for dauer arrest [9], in these survival phenotypes. Reducing protein synthesis in *daf-16(mgDf47)* mutants still causes developmental arrest (S3A Fig) and results in increased resistance to oxidative (S3B Fig) and thermal (S3C Fig) stress. We further note that these animals are not dauers, morphologically (S1F Fig) and are not resistant to treatment with 1% SDS—a phenotype of animals that successfully enter dauer diapause. Moreover, reducing protein synthesis in *daf-2(e1368)* mutants, which form constitutive dauers at the restrictive temperature of 25C, enter this L2 arrest stage instead of developing into dauers. These findings support the protein synthesis inhibition arrest state at the L2 larval stage and prior to dauer formation, which is an alternative L3 stage (S3D Fig).

### The hypodermis can mediate systemic responses during protein synthesis inhibition

**Fig 2:**
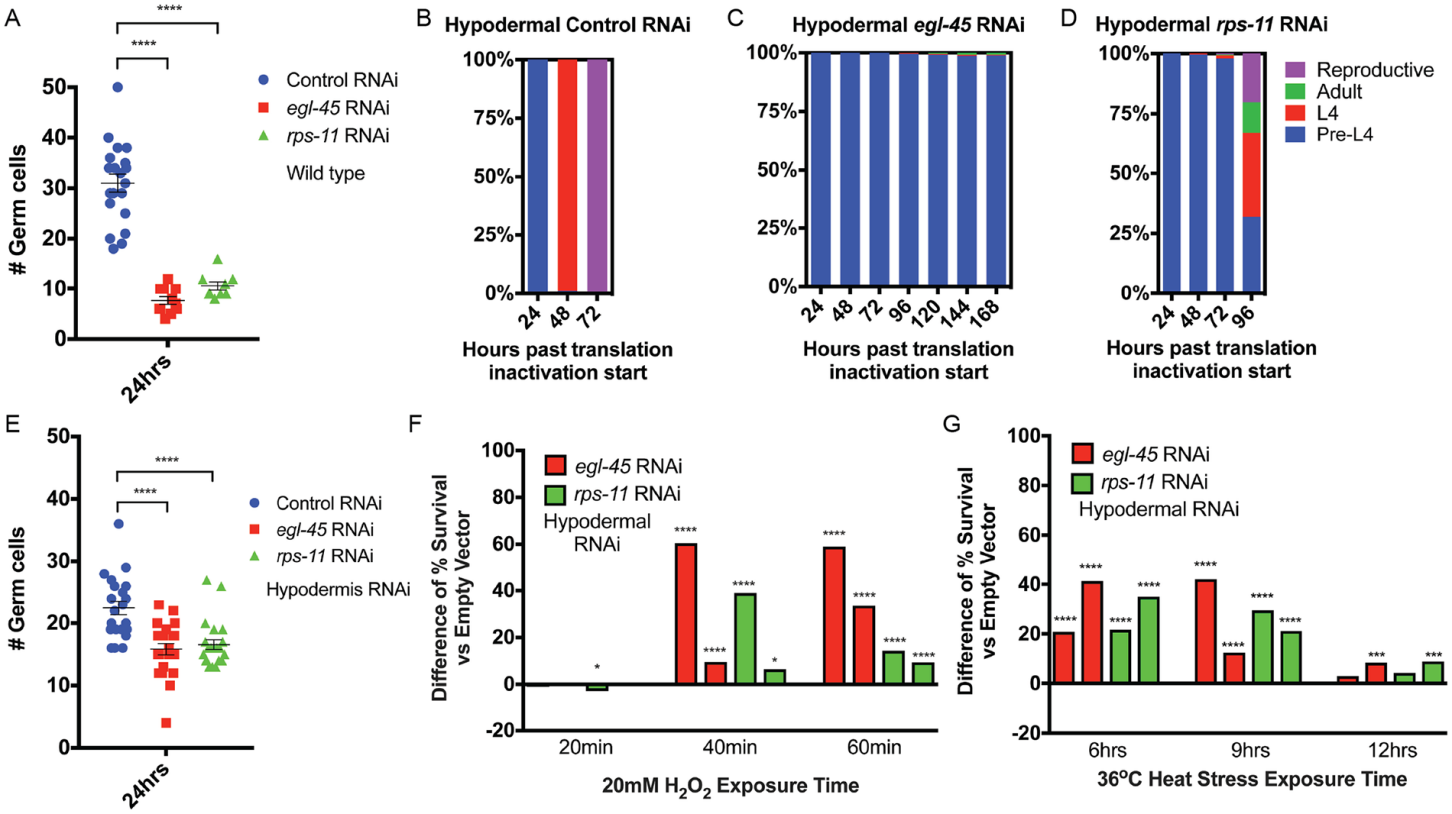
Cell autonomous and cell non-autonomous pathways contribute to organismal responses to protein synthesis inhibition. *A*. Germ cell proliferation is restricted in response to protein synthesis inhibition (N = 9–20). *B*-*D*. As compared to control RNAi (*B*), hypodermal-specific RNAi targeting *egl-45* (*C*) or *rps-11* (*D*) is sufficient to drive developmental arrest; pre-L4 (blue) to L4 (red), to adult (green), and reproductive adult (purple). Note that "pre-L4" is almost exclusively L2 stage past 24hrs, based on germline and molting data (see Methods for details) (N = 279–335 from 2 biological replicates). *E*-*G*. Hypodermal-specific protein synthesis inhibition arrests germ cell proliferation (*E*) (N = 21–22 from 2 biological replicates), organism level resistance to oxidative (*F*) and thermal (*G*) stress as compared to control RNAi-treated L2 stage animals (the bars in each stress resistance graph represent a unique set of biological replicates (S1 Table) relative to its own independent control cohort, N = 178–403 from 2–3 biological replicates). * p<0.025, ** p<0.005, **** p<0.00005 (*A*, *E*: One-way ANOVA; *F*-*G*: Fisher's exact test). See also S1 Table.

Considering the need for every cell to sense and respond to changes in biosynthetic capacity, but also the benefit of coordinating a systemic physiological response to a perceived organism-level deficit in any tissue, we hypothesized that the response to protein synthesis inhibition would be both cell autonomous and non-autonomous. The germline is a facile model for cell division in early larval development in *C*. *elegans* [34]. Similar to the developmental arrest observed at the organism level, tissue-general protein synthesis inhibition resulted in the clear arrest of the reproductive tissue at a stage typical for L2 animal development (Fig 2A). We next sought to determine which tissues were capable of initiating the L2 arrest. Using tissue-specific RNAi, we systematically reduced the expression of *egl-45*/*EIF3* or *rps-11*/*RPS11* in the intestine, germline, or hypodermis (S4A Fig). Similar to tissue-general RNAi, hypodermal-specific protein synthesis inhibition induced potent developmental arrest (Fig 2B–2D) and halted germline proliferation (Fig 2E). In contrast, while still slowing development, intestinal or germline-specific RNAi was unable to induce developmental arrest (S4B–S4J Fig). Germline-specific protein synthesis inhibition results in sterility (S4H–S4K Fig), which differentiates the cell autonomous effects of protein synthesis inhibition from the cell non-autonomous impact on the entire organism when diminished biosynthetic capacity is restricted to the hypodermis. Hypodermal-specific protein synthesis inhibition was the most effective at enhancing resistance to oxidative (Fig 2F) and thermal (Fig 2G) stress, as compared to germline- and intestine-specific RNAi (S4M–S4P Fig), which had modest or no effect on stress resistance. Moreover, hypodermal-specific protein synthesis inhibition initiated post-developmentally was capable of increasing lifespan and, in the case of *egl-45* RNAi, was at least equally potent as tissue-general protein synthesis inhibition (S4Q Fig). As predicted by their essential roles in protein synthesis, *egl-45/EIF3* and *rps-11/RPS11* expression is detectable in several tissues (S4R–S4U Fig), but the differences in the expression level and location could explain the variance in the strengths of phenotypes observed in *egl-45* RNAi versus *rps-11* RNAi. Nevertheless, these data identify the hypodermis as an important mediator of organismal regulation of growth and development in response to diminished biosynthetic capacity.

### Protein synthesis inhibition increases organismal Ca^2+^ levels and induces *mtl-1* expression

We examined the transcript levels of a panel of genes with established roles in stress adaptation (see Methods) under both 24 hours and 120 hours exposure to protein synthesis inhibition (collected after 24 and 120 hour exposure to RNAi) [35]. Despite the enhanced stress resistance observed in protein synthesis inhibition-induced L2 arrested animals, the expression of most genes tested—including several heat shock proteins, redox homeostasis pathway components, and isoforms of superoxide dismutase—was significantly repressed (S5A–S5J Fig).

**Fig 3:**
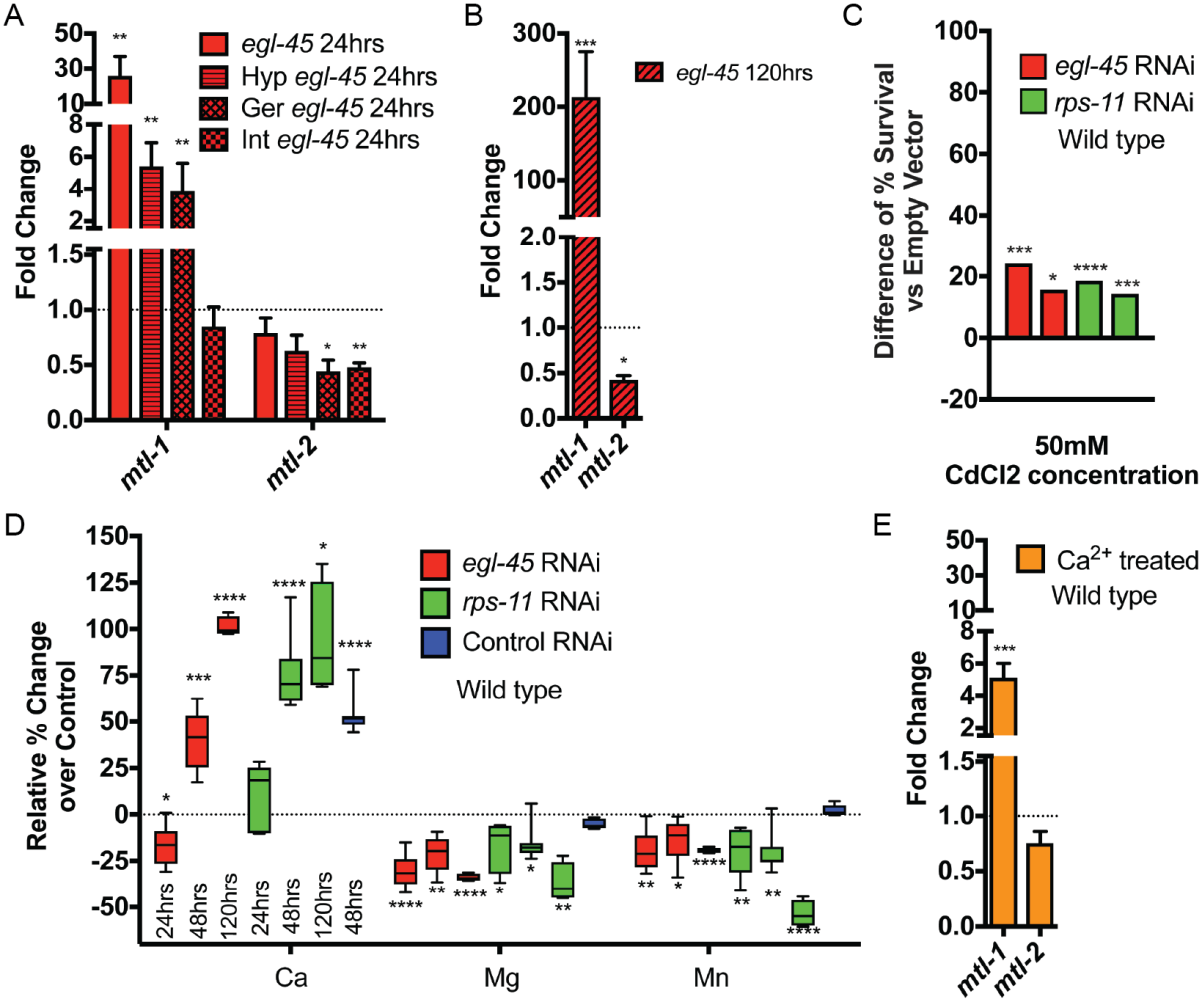
Protein synthesis inhibition induces *mtl-1* expression in response to deregulated Ca^2+^ homeostasis. (*A*-*B*) Organismal expression of *mtl-1* is increased while *mtl-2* is decreased in response to protein synthesis inhibition generated by RNAi in all cells (solid bars), hypodermal-specific RNAi (Hyp), or the germline-specific RNAi (Ger), but not the intestine-specific RNAi (Int) (*A*); this expression is more pronounced after 5 days (120hr) in the arrested state (*B*) (3 biological replicates). *C*. Protein synthesis inhibition-induced arrested L2 larvae are resistant to toxic levels of Cd^2+^ (50mM) (N = 188–251 from 2 biological replicates). *D*. Quantification of ICP-AES analysis of steady state metal levels in protein synthesis inhibition treated wild type animals. Samples are compared to 24-hour old, L2 stage, animals fed Control RNAi (from 4–7 biological replicates). *E*. Wild type animals exposed to 500mM CaCl_2_ induce the expression of *mtl-1* similar to protein synthesis inhibition treated animals (3 biological replicates). * p<0.05, ** p<0.01, *** p<0.001, **** p<0.0001 (*A-B*, *E*: Student's t test). * p< 0.025, ** p<0.005, *** p<0.0005, **** p<0.00005 (*D*-*E*: Student's t test; *C*: Fisher's exact test). See also S1 Table.

The notable exception in this panel was the expression of *mtl-1*, a metallothionine involved in metal homeostasis, which after 24 hours of either *egl-45*/*EIF3* or *rps-11/RPS11* RNAi was increased >10-fold (Fig 3A and S5E Fig); in animals arrested for 5 days, *mtl-1* was increased >100-fold (Fig 3B and S5F Fig). This temporal enhancement was not observed for other genes involved in stress adaptation (S5G–S5J Fig). Moreover, hypodermal-specific protein synthesis inhibition also induced *mtl-1* expression (Fig 3A and S5E Fig), consistent with the notion that the hypodermis is a potent sensor for organismal biosynthetic capacity. As *mtl-1* is activated in response to heavy metals, we challenged protein synthesis inhibition-arrested animals to toxic levels of Cd^2+^ (50mM) and discovered this arrest state also enhanced resistance to heavy metal stress (Fig 3C). Because heavy metal resistance was not previously annotated in adults with protein synthesis inhibition [19–21], we initiated protein synthesis inhibition post-developmentally by *egl-45/EIF3A* or *rps-11/RPS11* RNAi, which also resulted in resistance to Cd^2+^ exposure (S5K Fig). Similar to oxidative and thermal stress, hypodermal-specific RNAi of *egl-45/EIF3* or *rps-11/RPS11* could recapitulate the whole animal RNAi phenotype (S5L Fig). We next tested whether the increase in *mtl-1* was causative for the resistance, so we created a double mutant of *mtl-1(tm1770)* and *mtl-2(gk125)* (*mtl-2* is a related metallothionine also activated in response to heavy metals), which greatly attenuated the ability to survive Cd^2+^ exposure when protein synthesis is inhibited (S5M Fig). Based on these heavy metal responses, we wanted to further test if hypodermal RNAi could increase *mtl-1* to the same degree as observed in wild type animals exposed to protein synthesis inhibition for extended periods. Correlating with the rate of developmental arrest, *mtl-1* levels increase out to 48 and 120hrs of exposure to hypodermal specific RNAi of *egl-45* or *rps-11* (S5N Fig). However, animals with longer exposure to *rps-11* RNAi have *mtl-1* transcript levels that return to near wild type levels, which correlates with the escape from developmental arrest under hypodermal specific *rps-11* RNAi (Fig 2D).

Although heavy metals are not abundant in standard growth media, these findings led us to examine the total metal content of animals in protein synthesis inhibition arrest by inductively coupled plasma-atomic emission spectroscopy (ICP-AES). The metal profiles revealed a significant reduction in Mg^2+^ and Mn^2+^ and a marked increase in Ca^2+^ (Fig 3D and S6A Fig). These steady-state concentrations of metals were maintained in animals trapped in the arrested state for 5 days (Fig 3D and S6A Fig). *mtl-1;mtl-2* double mutant animals reduced multiple metal species by 10–20%, but did not affect Ca^2+^ levels (S6B–S6D Fig); protein synthesis inhibition treatment in this mutant was still able to induce many of the same Mg^2+^, Mn^2+^, and Ca^2+^ changes as seen in wild type, consistent with the transcriptional induction of *mtl-1* acting as a stress response rather than as the upstream effector. Moreover, animals acutely exposed to CaCl_2_ treatment as larvae have an *mtl-1* transcriptional profile that mirrors animals with protein synthesis inhibition (Fig 3E), suggesting that the increase in Ca^2+^ could be physiologically significant and promote the increased *mtl-1* expression.

### Protein synthesis inhibition reduces pharyngeal pumping in developmentally arrested and adult *C*. *elegans*

**Fig 4:**
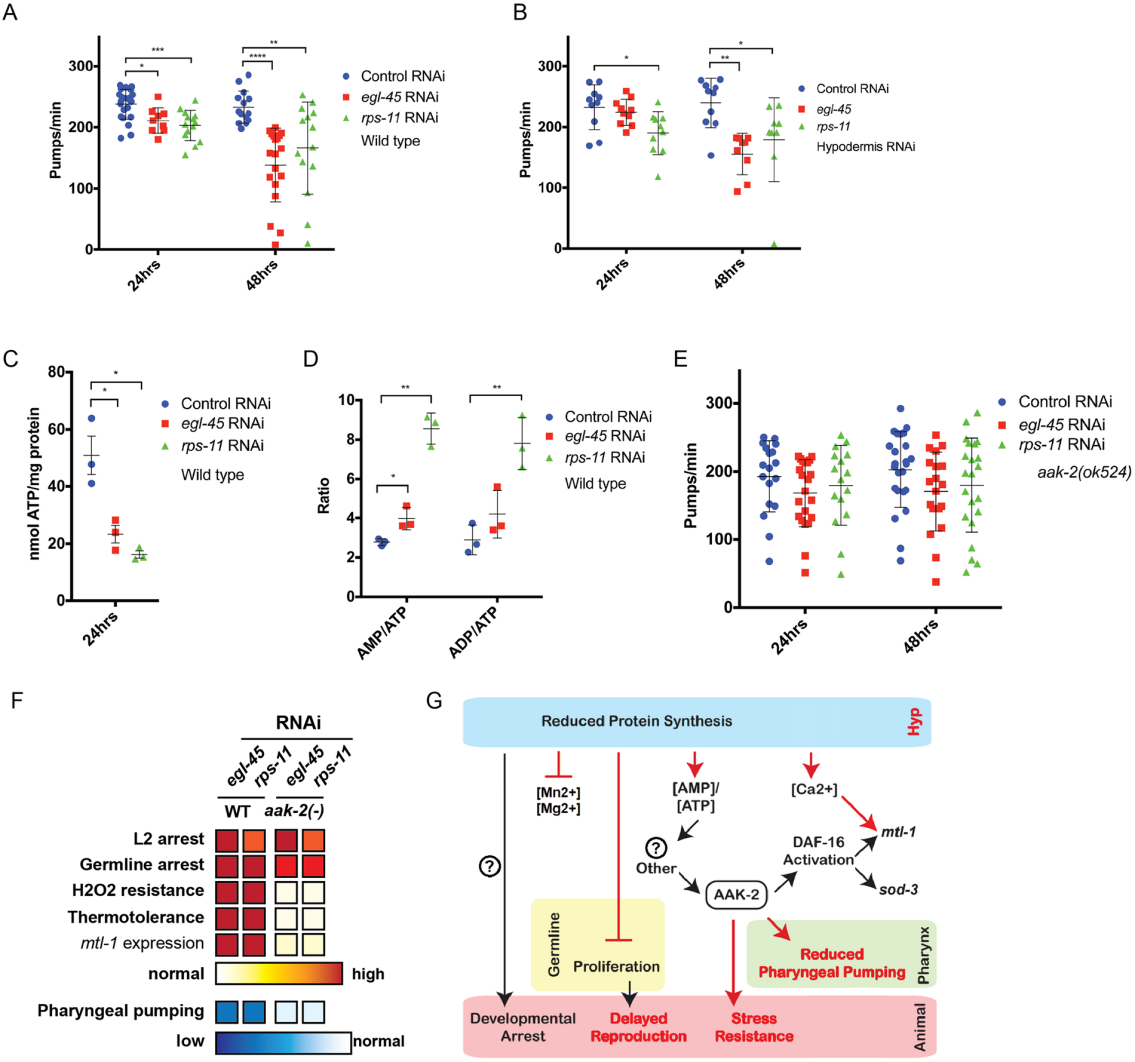
Protein synthesis inhibition drives a reduction in pharyngeal pumping and stress resistance through AMPK signaling. *A*. Protein synthesis inhibition decreases pharyngeal pumping rates in wildtype animals in a time-dependent manner (N = 9–23 from 2–3 biological replicates). *B*. Reducing protein synthesis in the hypodermis is sufficient to reduce pharyngeal pumping rate (N = 9–10 from 2 biological replicates). *C*-*D*. Protein synthesis inhibition arrested larvae have reduced levels of cellular ATP, and their ratio of AMP and ADP to ATP is higher (3 biological replicates). *E*-*F*. *aak-2/AMPK* mutant animals fail to reduce pharyngeal pumping in response to protein synthesis inhibition (*E*) and although arrested, are not resistant to environmental stress (*F*) (N = 16–24 from 2–3 biological replicates; intensity of red and blue coloring indicates increased and decreased responses, respectively, as compared to control RNAi treated animals). *G*. Schematic diagram of the cell autonomous and cell non-autonomous pathways that mediate organism-level responses to protein synthesis inhibition—red colors identify findings of this study. Additional mediators (?) are likely to exist, including the genetic regulators of the developmental arrest (left). * p<0.025, ** p<0.005, *** p<0.0005, **** p<0.00005 (*A*-*B*, *E*: One-way ANOVA, *C-D*: Student's t test). See also S1 Table.

Animals have adopted several strategies, ranging from molecular adaptation to changes in behavior, in order to cope with less than ideal growth conditions [36], and calcium plays several critical functions in these physiological responses. As such, we examined the behaviors of animals arrested from protein synthesis inhibition and noted a marked decrease in pharyngeal pumping (Fig 4A and S7A Fig), a rhythmic behavior influenced by calcium transients [37, 38]. The reduction in pharyngeal pumping was significant after 24-hours of protein synthesis inhibition and was more pronounced the more time animals were in the arrested state (S7B Fig); despite this reduction, a basal level of pumping continues even after 15 days in the arrested state (S7B Fig). Similar to the developmental arrest and enhanced stress resistance observed in *daf-16(mgDf47)* animals, *daf-16* is not required for the reduction in pharyngeal pumping rates when protein synthesis is inhibited (S7C Fig). In line with previous cell non-autonomous effects, hypodermal-specific protein synthesis inhibition effectively reduced pharyngeal pumping (Fig 4B), while protein synthesis inhibition in other somatic tissues could not evoke the same magnitude of responses (S7D and S7E Fig). This reduction of pharyngeal pumping is intriguing as this behavior is correlated with food intake [39], and caloric-restriction (CR) is an established means of enhancing organismal health- and lifespan [40, 41]. With this in mind, we measured pharyngeal pumping in adult worms fed *egl-45* or *rps-11* RNAi to induce protein synthesis inhibition, which are long-lived [16], and also discovered a significant reduction in pharyngeal pumping (S7F Fig). Taken together, these data define reduced pharyngeal pumping as a physiological response of protein synthesis inhibition during development and adulthood.

### AMPK signaling mediates the survival benefits of developmental protein synthesis inhibition

Protein synthesis is energetically expensive, and it is possible that protein synthesis inhibition leads to a state of excess ATP, which could be redirected to other cytoprotective pathways that drive stress resistance [42]. However, we found that animals exposed to protein synthesis inhibition during development have 50% less cellular ATP (Fig 4C). AAK-2/AMPK is a conserved sensor of energy homeostasis that responds to changes in cellular AMP/ATP levels [43]. Indeed, animals with protein synthesis inhibition have significantly higher AMP/ATP and ADP/ATP ratios (Fig 4D). As such, we tested *aak-2* mutants for the protein synthesis inhibition survival and arrest phenotypes. *aak-2(ok524)* mutants exposed to protein synthesis inhibition were still arrested as L2 animals with reduced germ cell counts (S8A–S8C Fig), but failed to dampen pharyngeal pumping rates (Fig 4E and S8D Fig), which importantly uncouples these two protein synthesis inhibition responses and suggests that the developmental phenotypes are not a result of diminished food intake. Additionally, *aak*-2 mutant animals failed to evoke protein synthesis inhibition responses observed in wild type animals (Fig 4F). Specifically, *aak-2* mutants have minimal, often undetectable, changes in the expression of *mtl-1* during protein synthesis inhibition (S8F Fig)—a phenotype similar to *daf-16* mutant animals (S8G Fig), which is a known regulator of the *mtl-1* locus (S8H Fig). *aak-2* mutants are also as sensitive to Cd^2+^ as wild type animals (S8I Fig), which further supports the connection between *mtl-1* expression with resistance to environmental metal exposure. Furthermore, *aak-2* mutants with protein synthesis inhibition are as sensitive to oxidative and thermal stress as wild type animals (S8J, S8K, S8M and S8N Fig), indicating the essentiality of AMPK signaling in protein synthesis inhibition-induced stress resistance.

We then tested mutant animals harboring a truncated and constitutively active (CA) form of AAK-2 [44], which slowed development [44] and afforded resistance to oxidative stress while restoring thermal stress resistance under reduced protein synthesis, relative to *aak-2* mutants (S8A, S8C, S8J, S8L, S8M and S8O Fig). Intriguingly, expression of a constitutively activated version of AMPK (CA-AMPK [44]) restored the reduction of pharyngeal pumping phenotype when protein synthesis was reduced (S8D and S8E Fig). Taken together with the AMP/ATP and ADP/ATP levels (Fig 4G), these data define an AAK-2/AMPK molecular pathway that initiates organismal-level physiological responses to cellular deficiencies in protein synthesis. Importantly, our studies reveal a clear role for AMPK signaling in mediating the survival responses to protein synthesis inhibition beyond developmental arrest.

### Developmental exposure to microorganisms producing protein synthesis inhibition-inducing xenobiotics recapitulate the arrest-survival state

**Fig 5:**
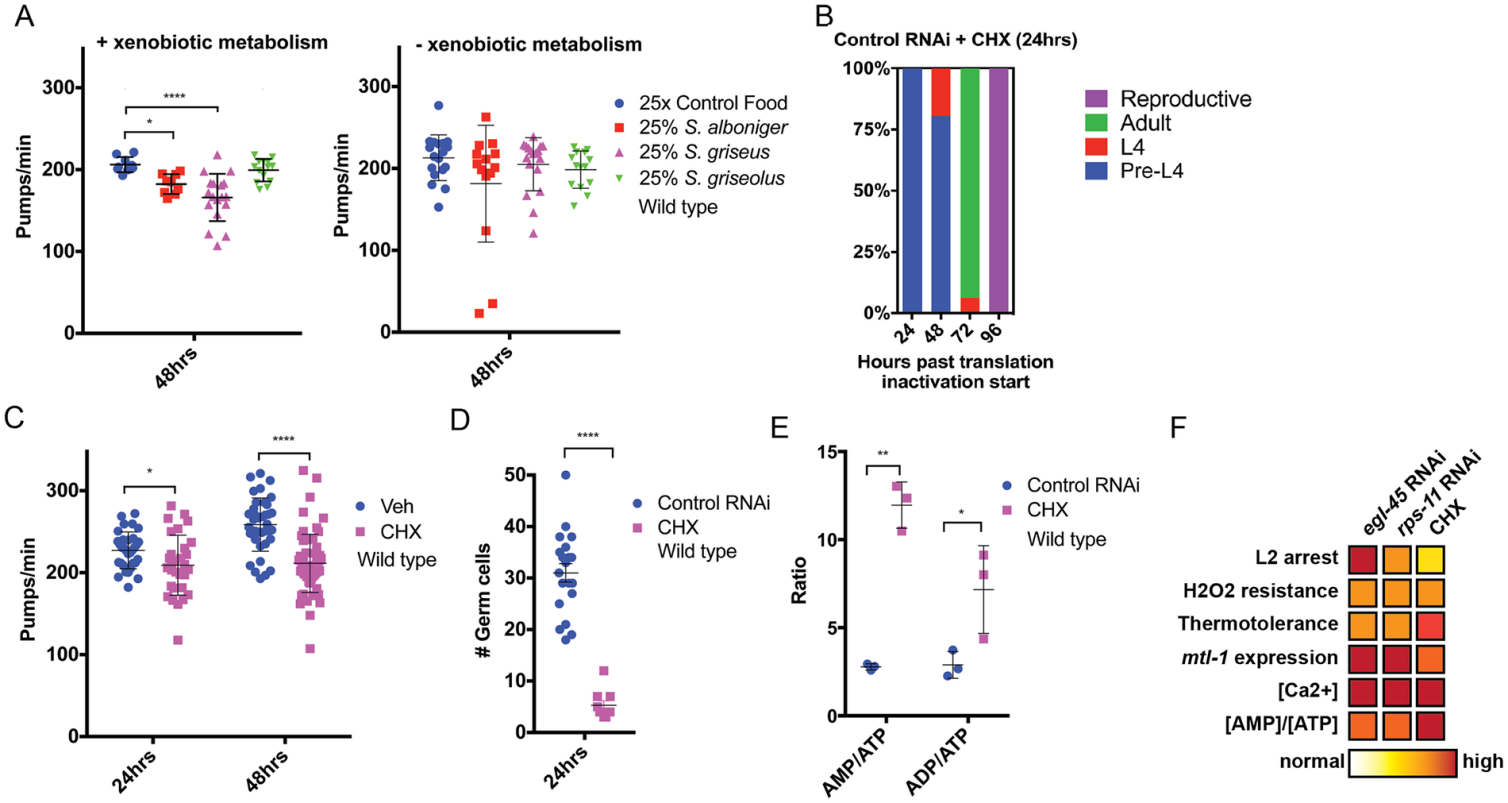
*Exposure to the* xenobiotic cycloheximide mimics the survival responses to transcriptional and genetic reduction of protein synthesis. *A*. *C*. *elegans* exposed to *Streptomyces* species, that generate xenobiotics targeting eukaryotic protein synthesis, reduce pharyngeal pumping, but not when secondary metabolism is repressed (N = 9–18 from 2 biological replicates). *B*. Animals exposed to 0.05mg/ml CHX for 24hrs, then removed to vehicle, can fully recover to reproduction (N = 93 from 2 biological replicates). *C*-*E*. Treatment of *C*. *elegans* with cycloheximide (CHX) reduces pharyngeal pumping (*C*) (N = 31–64 from 3 biological replicates), arrests germ cell proliferation (*D*) (N = 10–20), increases the ratio of AMP and ADP to ATP (*E*) (3 biological replicates), and induces protein synthesis inhibition developmental arrest and stress resistance phenotypes; intensity of red coloring indicates increased responses as compared to control RNAi (*F*). * p<0.0166, **** p<0.000033 (*A*: One-way ANOVA); * p<0.05, ** p<0.01, **** p<0.0001 (*C*-*E*: Student's t test). See also S1 Table.

In the context of a worm’s natural environment, we postulated that the ability to pause development in response to a perceived cellular deficiency would be advantageous—and perhaps evolved—as a response mechanism to deal with environmental hazards. In the wild, *C*. *elegans* consume diets that are far more complex than the simple and homogenous *E*.*coli* lawn provided to them in the laboratory [1]. These wild diets include heterogeneous populations of microorganisms, some of which can produce xenobiotic compounds that can target and disable essential biological pathways. Recently, the soil and intestinal microbiome of *C*. *elegans* has been characterized [45–47]. While only appearing at rates ranging from 0.001–0.1% in soil samples found in these studies, we chose to focus on the genus *Streptomyces*, as it is soil-dwelling, readily accessible with the lowest biosafety level, and has several members that produce commonly utilized molecules that can potently inhibit eukaryotic protein synthesis [48]. If wild *C*. *elegans* came upon a microcosm of *Streptomyces* species, or any other organism capable of producing xenobiotics that reduce protein synthesis, it would be important to have defenses available against these molecules. We exposed worms to *S*. *griseus*, *S*. *griseolus*, or *S*. *alboniger*, that produce cycloheximide (CHX), anisomycin, and puromycin, respectively (S9A Fig). Exposure to these *Streptomyces* species grown under stationary conditions for five days, in order to initiate secondary metabolism and the creation of these protein synthesis inhibition molecules [49], resulted in delayed reproduction (S9B Fig) and significant reduction of their pharyngeal pumping in two species (Fig 5A). This is in contrast to exposure with microbes in exponential phase growth which attenuates secondary metabolism [49] (Fig 5A).

Exposure to pathogens can alter several physiological parameters in the host, and of all the pathogens tested, exposure to *S*. *griseus* exerted the strongest influence on pharyngeal pumping. The remarkably similar impact that exposure to *S*. *griseus* had on *C*. *elegans* development and physiology, as compared to RNAi-induced protein synthesis inhibition, drove a further examination of how exposure to cycloheximide (CHX), the bioactive secondary metabolite produced by *S*. *griseus*, affected *C*. *elegans* survival during development. CHX is a potent inhibitor of ribosome processivity and has recently been shown to exert health-promoting effects in adult *C*. *elegans* by an unknown mechanism [50].

Satisfyingly, CHX exposure upon hatching, which inhibits new protein synthesis (S9C Fig), also resulted in arrested animal development (S9D Fig and Fig 5B), and can arrest in a dose-dependent manner (S9D Fig). Although animals arrested by RNAi-mediated protein synthesis inhibition can continue development upon removal from the RNAi state, not all animals in the population mature into fertile adults (S9E–S9G Fig)—likely a result of the persistence of RNAi [51–54]. However, initiating protein synthesis inhibition *via* exposure to 0.05mg/ml CHX rather than RNAi of essential protein synthesis factors (S1A Fig) enabled studies of recovery from the arrest state without the complications of RNAi. Once removed from the xenobiotic, developmentally arrested animals resume development—indicating the arrest state is truly transient (Fig 5B, S2 Table). The CHX-induced arrest state caused reduced pharyngeal pumping (Fig 5C), arrested germ cell proliferation (Fig 5D), increased organismal [AMP]/[ATP] ratio (Fig 5E). Importantly, this arrest state phenocopied all RNAi-based protein synthesis inhibition survival responses (Fig 5F) including: enhanced resistance to oxidative (S9H Fig) and thermal stress (S9I Fig), induced the expression of *mtl-1* (S9J Fig) decreased cellular ATP (S9K Fig), and resulted in metal profiles similar to animals fed RNAi targeting *egl-45*/*EIF3* and *rps-11*/*RPS11* (S9L and S9M Fig). 0.05mg/ml CHX exposure may not fully arrest all animals, as some *daf-2(e1368)* animals at the restrictive temperature did become dauers (S2Q Fig). Animals that are released from CHX arrest have minimal (if any) changes in reproductive output (S9N Fig), have a small but significant increase in resistance of oxidative stress (S9O Fig), are delayed ~16-20hrs to reproduction (S9P Fig), and have normal pumping rates at physiological day 3 of adulthood (S9Q Fig). Thus, this transient arrest state is survival promoting when the deficiency in protein synthesis is present and is not afforded once homeostasis is reestablished, similar to animals released from dauer [36]. Intriguingly, the ability of *Streptomyces griseus* to reduce pharyngeal muscle pumping required the presence of live bacteria co-culture (S9R Fig). In addition, increasing doses of CHX, similar to the threshold effects seen with RNAi targeting genes involved in protein synthesis (S1E Fig), could further reduce the pumping rate of the arrested animal (S9S Fig). Thus, the complexity of the environment and drug dosage are important for balancing the induction of this survival state.

## Discussion

In response to impaired organismal protein synthesis, animals are capable of entering an arrest state, reaping survival benefits, and exiting to become reproductive adults (Fig 5B). In our studies, we are forcing continual exposure of animals to protein synthesis inhibiting RNAi or xenobiotics, which is likely "unnatural", as previous studies of lethal RNAi treatment and xenobiotic treatments leads to aversion behaviors [15, 17]. With this in mind, we predict that in the wild the perceived loss of translation would evoke a similar aversion response—allowing animals to escape to new pathogen-free environments. This model is supported by our studies with cycloheximide exposure, which drives a rapid induction of arrest and stress resistance, from which animals can quickly recover. In this regard, we believe that the use of cycloheximide as a transient inducer of protein synthesis inhibition in the worm will be of great use in studying protein synthesis inhibition going forward in order to circumvent the complications of RNAi expansion over the worm lifespan and subsequent generations. Given that there is a dose response to CHX exposure, higher doses can be utilized to prolong the arrest state and enhance arrest phenotypes although prolonged exposure to higher concentration reduces the rate of escape (S2 Table).

The lack of necessity of DAF-16 for the developmental arrest in response to protein synthesis inhibition indicates that the reduced protein synthesis pathway functions independently from the dauer development pathway. Yet, while most dauer constitutive *daf-2* mutants that are arrested from CHX do not form dauers, intriguingly ~20–25% will develop into dauers instead of undergoing protein synthesis arrest (S3D Fig). This finding suggests that animals can either alternatively arrest in the L2d stage [33, 55, 56], or that the CHX dose requires a higher threshold for complete arrest of animals (especially given the 100% non-dauer RNAi-treated animals). Of note, reduced protein synthesis arrested animals are distinct from the L2d stage as they are of smaller length than wild type L2s (S1G Fig) (unlike 50% longer L2d animals [33]), functional AMPK is not necessary for the reduction of germ cell numbers (S8B Fig) as it is in L2d/dauer animals [57], and we have never observed them becoming dauers after exiting the arrest state. Future characterization of any phenotypic parallels between L2d and reduced protein synthesis arrest, especially in the context of the differing role of AMPK in controlling germ cell proliferation, will be of interest for future studies.

A persistent question in biology asks how cellular status is communicated across the organism and, more importantly, how an appropriate homeostatic response is engaged. Protein synthesis inhibition in the hypodermis alone was sufficient for all arrest and healthspan phenotypes. In addition to its important role in the molting process during larval development, the hypodermis has recently been implicated as being important in dietary checkpoints in larval arrest [9, 58]. Although it is known that *C*. *elegans* tissues have differential capacity for RNAi, our work bolsters the hypodermis as a key tissue in larval development, and identifies a new cell non-autonomous communication pathway to initiate systemic responses. Given that the hypodermis is the first barrier to its external environment that covers the entire organism, it is reasonable that *C*. *elegans* might evolve sensing mechanisms for hypodermal cellular changes to influence whole-body cellular signaling. It is also possible that the high demand for protein synthesis during growth of the developing hypodermis amplifies the tissue-general effects of protein synthesis inhibition in this tissue, with or without specifically evolved signaling pathways. However, proliferation alone is not the only factor that influences responses to protein synthesis inhibition. The germline is a highly proliferative tissue in *C*. *elegans*, and while protein synthesis inhibition in the germline did not result in the same L2 arrest state as tissue-general or hypodermis-specific reduction, it did result in pre-reproductive adult animals with mild stress resistance (S4 Fig). It remains to be seen if this germline arrest is also reversible, similar to starvation-induced adult reproductive diapause [4].

It is important to note the differences in stress resistance when protein synthesis is reduced in specific tissues. While hypodermis-specific RNAi of protein synthesis components results in increased stress resistance that is consistent when RNAi is initiated in all tissues, intestine-specific RNAi resulted in no change to stress resistance capacity except for a few instances of increased resistance only observed for *rps-11* RNAi. The more tissue-general expression of *rps-11*/*RPS11* (S4 Fig), may explain these minor phenotypic differences as compared to *egl-45/EIF3* RNAi. Taken together, these data support the idea that the systemic stress responses that stem from the loss of *rps-11* are mediated by effects across multiple tissues. In contrast to the hypodermis and intestine, germline-specific loss of protein synthesis resulted in modest or no changes in oxidative stress resistance and surprisingly lead to reduced thermal tolerance. This suggests that the oxidative and thermal stress resistance responses, at least in the germline, may be uncoupled or, alternatively, that reducing protein synthesis in the germline activates a separate pathway that negatively affects thermal stress resistance. Finally, it is also worth noting that there is considerable variation in stress resistance among these tissue-specific RNAi strains. We attribute much of this both to the use of RNAi variance, as well as the ever-present "leakiness" of these tissue specific strains that can sometimes spread RNAi effects to other tissues [59, 60].

The metallothionein, *mtl-1*, is highly (>100-fold) upregulated under reduced protein synthesis. The increased expression of *mtl-1* was required for heavy metal resistance in animals with protein synthesis inhibition, which is notable since hypersensitivity to cadmium has not been reported in adult *C*. *elegans* lacking MTL-1 or MTL-2 [61]. This finding further advocates for the importance of uncoupling developmental and adult specific responses. Transcription of *MT1*, the mammalian homolog of *mtl-1*, is also upregulated by oxidative stress agents in cell lines and mice [62, 63], so it is possible that protein synthesis inhibition causes an increase in ROS that triggers *mtl-1* transcription; however, then we would also expect to see increased transcription of SKN-1 target genes (e.g. *gst-4*), which we do not observe. Moreover, *mtl-1* expression was not necessary for the arrest, oxidative or thermal stress resistance, or reduced pumping, as *daf-16* mutants (which lack *mtl-1* expression, S8 Fig) still display both phenotypes. Thus, given the very specific transcription of *mtl-1*, the changes in expression are likely due to the presence of its most well-defined binding partners, metal cations. Traditional targets of MTL-1 are Zn^2+^, Cd^2+^, and Cu^2+^, but mammalian homologs can bind to Mg^2+^, Mn^2+^, and Ca^2+^ [64–66]. The increase in Ca^2+^ ions could be the cause of this high transcriptional response, especially given that Ca^2+^ treatment could induce *mtl-1* in worms (Fig 3E). However, it is also possible that higher levels of other heavy metals, such as Cd^2+^, which never reached our detection limits, are responsible. Given that *mtl-1* expression was disposable for the arrest, stress resistance, and reduced pumping rate, the increased expression change is a "biomarker" for the reduction of protein synthesis, rather than a central player in this developmental state. Given the ability for calcium to upregulate this *mtl-1* response (Fig 3), we expect the protein synthesis loss triggers calcium abundance and *daf-16* activation [16], that both go on to increase *mtl-1* levels.

It is possible that the reduction of cellular ATP we observe reflects the use of ATP to “power” survival processes [42]. However, a ~50% reduction in ATP after 24hrs of protein synthesis inhibition is a remarkable loss, and it would not explain how this energy usage would be sustained to continue stress resistance over extended time periods, especially when accompanied by a reduction in pharyngeal pumping (thereby reducing food/energy intake even further). Our data support an alternative model where increases in the [AMP]/[ATP] and [ADP]/[ATP] ratios activate AMPK pathways that signal for downstream survival pathways (Fig 4C and 4F). The underlying mechanism driving the imbalance to cellular adenylate pools will be of future interest.

We found that AMPK was necessary for all of our protein synthesis inhibition survival phenotypes, except for arrest. AMPK activation has been implicated in survival phenotypes before, including glucose restriction pathways [67] and oxidative stress resistance [68] in *C*. *elegans*. Juxtaposed to our work, activating AMPK (such as via AICA ribonucleotide) causes a decrease in protein synthesis [23–25]. While our work focuses directly on protein synthesis alone, AMPK is also increased in *rsks-1*/S6K mutants [27, 28] and under starvation conditions [26]. This suggests that AMPK and protein synthesis may work together in a circular pathway or that they affect each other by cell non-autonomous signaling. In addition, an upstream activator of AMPK, ARGK-1, is both important for *rsks-1*/S6K mutant longevity, and its overexpression caused reduced pumping rates in worms [69]; further study into the role of ARGK-1 in this protein synthesis inhibition survival state will be of interest in future studies. As a final note, *C*. *elegans* lacking the elongation factor *efk-1*, which is activated by AMPK, fare worse under nutrient starvation conditions [70]; thus, there are multiple connections between starvation, protein synthesis, and energy homeostasis, and understanding them in context of survival states is important to consider.

Previous studies suggest that the effects of protein synthesis inhibition on adult lifespan are distinct from caloric restriction (CR) [19] and that the CR state can drive a reduction in protein synthesis[20]. Our data suggest that during development the opposite is also true: that protein synthesis inhibition can reduce pharyngeal pumping leading to a CR-like state. CR across most organisms has both life- and healthspan promoting effects; however, the evolutionary basis of the CR response is unknown. One hypothesis generated from this study is that the physiological response to CR might stem from an ancient program to promote stress resistance when the presence of diminished biosynthetic capacity is perceived. Microorganisms such as *Streptomyces* provide a potential evolutionary explanation to a mechanism of a pathogen-derived CR pathway by engaging behavioral avoidance phenotypes toward toxin-producing pathogens [15]. It is important to note that *Streptomyces* was found at very low levels in recent studies looking at *C*. *elegans* soil samples [45–47]. Our xenobiotic experiments are not meant to emulate the wild environment, but to capture the interaction between the worm and a harmful species in the environment. It is altogether possible that there are areas (or times in history) where *Streptomyces*, or other species capable of inhibiting host protein synthesis, are a more common occurrence, demanding the need for such an arrest survival response documented here.

**Fig 6:**
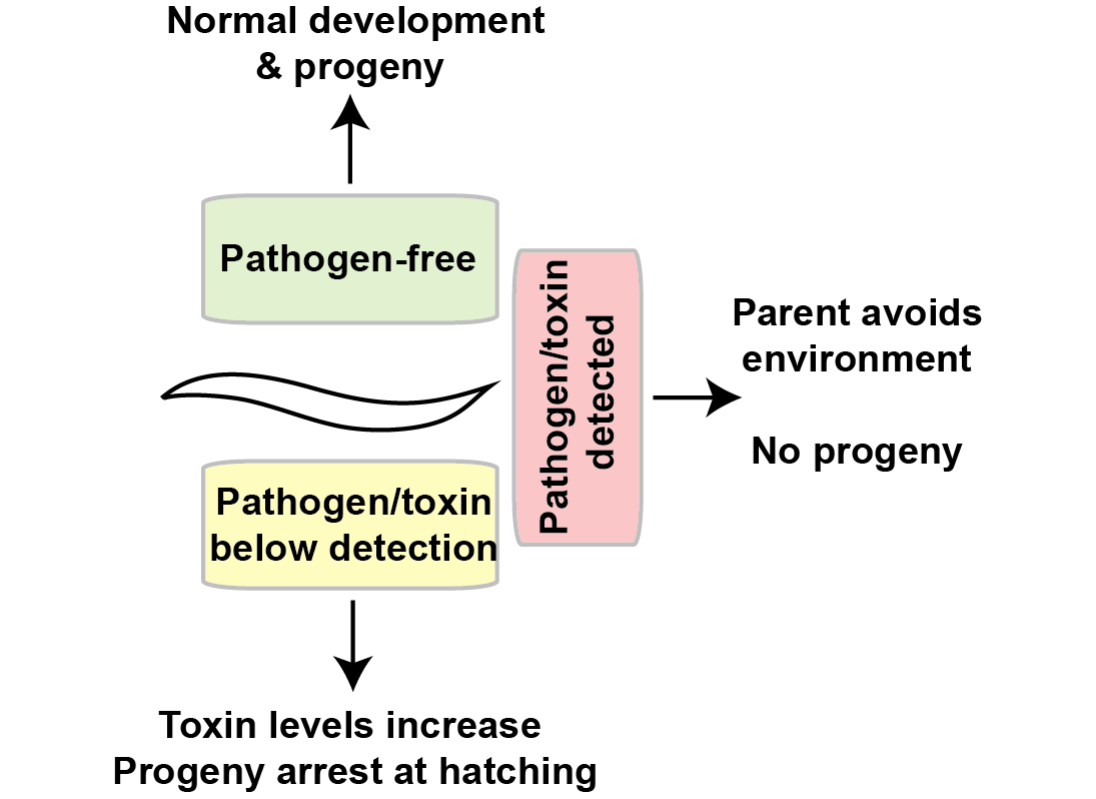
Schematic model of fitness-driven responses to toxin producing microbes. The ability of a worm to respond to toxins via stress responses, such as increasing stress resistance or decreasing pharyngeal pumping, is imperative to survive in a pathogen-rich environment.

There are connections between immune function and the regulation of protein synthesis—both to exposure to protein synthesis-impairing xenobiotics (ExoA, Hygromycin) as well as potential surveillance mechanisms for reduced protein translation as a surrogate for infection [71–73]. Pathogen response pathways can also be closely linked to promoting proteostasis [74]. In addition, a recent study found that *C*. *elegans* can enter a diapause to avoid pathogens (unlike our study, this is reliant on the formation of dauers [75]). Nevertheless, our findings support the idea that the loss of protein synthesis might be perceived as "an attack" by a pathogen, which initiates a reduction in pharyngeal pumping, that could minimize ingestion of toxin-producing microbes. Given the remarkable overlap in phenotypes resulting from protein synthesis inhibition by pathogen-derived xenobiotics and our genetic and RNAi-mediated protein synthesis inhibition, it is suggestive that this survival-arrest state could have evolved as a stress response to the presence of pathogens (Fig 6). This idea parallels models of adult longevity pathways, which may have connections to xenobiotics targeting other essential pathways besides protein synthesis [35].

Unlike previous models that suggest the developmental arrest resulting from early loss of protein synthesis is a detrimental state [42], these studies provide an alternative way of thinking about these developmental responses. The induction of protective responses to reduced protein synthesis is survival-promoting, and we predict that the capacity to engage these pathways would enable future opportunities for reproduction once the inhibition is alleviated. Lastly, our results provide an example of how the evolution and selection of developmental pro-fitness pathways may be utilized effectively later in life under the right conditions. Just as dauer diapause from reduced insulin/IGF-1 signaling (IIS) has mechanistic similarities with adult longevity responses when IIS is reduced post-developmentally, our studies establish a similar fitness-driven developmental program as the underlying mechanism of the enhanced healthy aging observed in adults with compromised protein biosynthetic capacity. The exceptional degree of conservation of these cellular pathways across organisms is suggestive that the pre- and post-developmental responses to protein synthesis inhibition observed in *C*. *elegans* could be similarly shared, even among humans.

## Methods

### *C*. *elegans* strains and culture

Worm strains were grown at 20°C for all experiments except dauer studies that were conducted at 25°C. All strains were unstarved for at least 3 generations (except for L1 synchronization) before being used in any experiments. List of strains used: N2 Bristol (wild type), DR1572 *daf-2(e1368)*, GR1329 *daf-16(mgDf47)*, MGH171 (*sid-1(qt9)*; Is[*vha-6*::*sid-1*::SL2::*gfp*], JM43 (*rde-1*(*ne219*); Is[*wrt-2p*::*rde-1*], *myo-2p*::*rfp*]), NL2098 (rrf-1(pk1417)), GR1395 (*mgIs49*[*mlt-10p*::*gfp-pest*, *ttx-3*::*gfp*]IV]), SPC365 *mtl-1(tm1770); mtl-2 (gk125)*, RB754 (*aak-2* (*ok524*)), SPC366 (*aak-2(ok524); uthIs248*[*aak-2p*::*aak-2*(genomic aa1-321)::*GFP*::*unc-54* 3'UTR (gain of function allele); *myo-2p*::*tdTOMATO*]), SPC363 (*Ex*[*egl-45p*::*rfp*; *rol-6(su1006)*]), SPC364 (*Ex*[*rps-11p*::*gfp; rol-6(su1006)*]), CL2070 (*dvIs70[hsp-16*.*2p*::*GFP; rol-6(su1006)])*, KX38 (*ifg-1*(*ok1211*)/mIn1 [mIs14 *dpy-10*(*e128*)]). Some strains were provided by the CGC, which is funded by NIH Office of Research Infrastructure Programs (P40 OD010440).

### Protein synthesis inhibition treatment by RNAi or xenobiotic

*E*. *coli* strain HT115 (DE3) containing empty vector L4440 (hereafter referred to as Control RNAi), or plasmid against a gene of interest, was grown overnight (16-18hrs) at 37°C and seeded on NGM plates containing 5mM isopropyl-β-D-thiogalactoside (IPTG) and 50ug/ml carbenicillin. The bacteria were allowed to generated dsRNA overnight before being used within the next 1–3 days (stored at 20°C for this period if not used immediately). Dose response curves were established by feeding HT115 bacteria expressing the indicated RNAi clone diluted with HT115 bacteria harboring the control RNAi plasmid L4440. 0.05mg/ml Cycloheximide (CHX) or water (vehicle control) was added on top of bacteria and allowed to dry and rest for at least 1 hour before placing worms on treated bacterial lawns; this was the concentration of CHX throughout this paper, unless otherwise noted.

Loss of protein synthesis was determined via measurements of *de novo* synthesis of GFP through both an internal (via natural development) and external (via high temperature) induction method. External: plated animals expressing *hsp-16*.*2p*::*GFP* were maintained at 20°C and fed RNAi since hatching. After 24hrs, one set of worms was shifted to 36°C for 3hr, while the other was mounted for the baseline 0hr time point. The baseline plate was also checked after 3 hours at room temperature as a control for any room temperature-induced GFP expression. Internal: plated animals expressing *mlt-10p*::*gfp-pest*, treated with RNAi or drug since hatching, were imaged via the same methods for GFP expression at 12, 14, and 16 hours post-feeding. Worms were imaged at 20x magnification with bright field and GFP filter (Zeiss Axio Imager).

### Protein synthesis arrest recovery

Plated animals, treated with drug or RNAi since hatching, were counted in 24 hours intervals via a compound microscope as larval stage 1–3 (size), larval stage 4 (vulval invagination), adult (size), or reproductive (presence of internal eggs). In food switching assays, worms were moved to *rde-1* RNAi after 24hrs on the listed RNAi. *rde-1* RNAi was used to inhibit the RNAi machinery because RNAi effects can persist even after moving animals off of food containing double stranded RNA for multiple generations.

### Thermotolerance

Plated animals, treated for 24 or 48 hours on drug or RNAi since hatching, were placed at 36°C for up to 12 hours. Every 3 hours, one set of plates was removed to room temperature. Worms were allowed to recover for at least 10 minutes, and then counted for survival immediately by checking for touch response to prodding with a platinum wire.

### Oxidative stress

Plated animals, treated for 24 or 48 hours on drug or RNAi since hatching, were washed with M9 buffer twice in microcentrifuge tubes, then treated with 20mM H_2_O_2_ for up to 1 hour while rocking at room temperature. Every 20 minutes, one set of worms was removed from rocking, washed 3 times in M9 buffer, and plated back onto new plates containing their previous treatment (drug or RNAi). Worms were checked 1 hour after plating to count any acute deaths ("straight line" bodies or ruptured vulvas) only by eye, and 24 hours after plating to count final survival as done in thermotolerance assay.

### Heavy metal stress

Plated animals, treated for 24 hours on RNAi since hatching or at L4/YA stage, were washed with K-medium (32mM KCl, 51mM NaCl in dH2O) twice in microcentrifuge tubes, then treated with 5 or 50mM CdCl_2_ in K-medium (hatched or YAs, respectively) for 30 minutes while rocking at room temperature. After 30 minutes, worms were washed 3 times in K-medium, and plated back onto new plates containing their previous treatment (RNAi). Worms were checked 1 hour after plating to count any acute deaths ("straight line" bodies or ruptured vulvas) only by eye, and 24 hours after plating to count final survival as done in thermotolerance assay.

### Dauer development assay

Wild type and *daf-2(e1368)* were placed as synchronized L1s onto the listed RNAi clone or drug at 25°C for 48hrs. Worms were then washed in M9, pelleted, and treated with 1% for 30min while rocked at room temperature. Treated animals were then plated onto plates with HT115 bacteria and counted for survival.

### qRT-PCR measurements

Drug- or RNAi-treated animals were washed with M9 buffer twice in microcentrifuge tubes, then frozen at -80°C in TRI-Reagent^®^ (Zymo Research, R2050-1-200). After at least 24 hours at -80°C, RNA was extracted from samples using the Direct-zol^™^ RNA MiniPrep kit (R2052). Quantitative reverse transcription PCR (qRT-PCR) was performed on the RNA samples with gene specific primers (Table 1).

**Table 1:** List of genes tested for expression and their qPCR primers.

*snb-1* F	CCGGATAAGACCATCTTGACG
*snb-1* R	GACGACTTCATCAACCTGAGC
*mtl-1* F	GCTTGCAAGTGTGACTGCAA
*mtl-1* R	TTTTTCTCACTGGCCTCCTC
*mtl-2* F	TCTGCAAGTGTGACTGCAAA
*mtl-2* R	CAGCAGTATTGCTCACAGCAC
*cdr-1* F	TCTTCTCTCAATTGGCAACTG
*cdr-1* R	TTTGGGTAAACTTCATGACGA
*gst-4* F	GATGCTCGTGCTCTTGCTG
*gst-4* R	CCGAATTGTTCTCCATCGAC
*hsp-4* F	CTAAGATCGAGATCGAGTCACTC
*hsp-4* R	GCTTCAATGTAGCACGGAAC
*hsp-6* F	TTAGAAACCCCCAACGTGTC
*hsp-6* R	CGGCACAAAGAACAGAACAA
*hsp-60* F	TCCAACTAAGGTGGTTCGC
*hsp-60* R	TGACTACGCATTCGGTTGTG
*hsp-70* F	TGAAAGAGAAGACGCAGCAC
*hsp-70* R	GCCTGCTTAACTTGGAATGC
*hsf-1* F	TTGACGACGACAAGCTTCCAGT
*hsf-1* R	AAAGCTTGCACCAGAATCATCCC
*daf-16* F	CTTCAAGCCAATGCCACTACC
*daf-16* R	GGAGATGAGTTGGATGTTGATAGC
*sod-2* F	TTTGGAAGATCGCCAACTG
*sod-2* R	TTGTGATTCAGCTCATTTATTGC
*ugt-11* F	CCGATTTCTGGGACTCTCAA
*ugt-11* R	GGACTCCCAGGAAGTGTGAC
*gcs-1* F	CCAATCGATTCCTTTGGAGA
*gcs-1* R	TCGACAATGTTGAAGCAAGC
*icl-1* F	TCTCCGTGGTATCCATGCC
*icl-1* R	TGATCGAAAACTCTCTTAGCC
*gpdh-1* F	GGAGCACTAAAGAACATTGTCG
*gpdh-1* R	GGATGATAGCGGATTTCACG
*sod-3* F	GCAATCTACTGCTCGCACTG
*sod-3* R	GCATGATGCTTTTGATGATGA
*sod-1* F	TTTTCCGCAGGTCGAAGC
*sod-1* R	CCTGGTCATTTTCGGACTTC
*sod-2* F	TTTGGAAGATCGCCAACTG
*sod-2* R	TTGTGATTCAGCTCATTTATTGC
*sod-4* F	TGGCCGAAGTGTGGTTATTC
*sod-4* R	TCAGACGGTACCGATAGTTCC
*sod-5* F	CTTCCACAGGACGTTGTTTCC
*sod-5* R	TGGGTAAGCCAAACAGTTCC

For evaluation of *mtl-1* induced by calcium, wild type animals, grown for 24 hours on Control RNAi, were washed with K-medium twice in microcentrifuge tubes and then treated with 500mM CaCl_2_ (in K-medium) for 30 minutes at room temperature. Animals were then washed three times with K-medium, frozen at -80°C in TRI-Reagent^®^ as above, and the same protocol as above was utilized.

### Developmental timing by *mlt-10p*::*gfp*

Two 24-well plates, each containing a single GR1395 worm on RNAi or Control RNAi, were visualized by fluorescence microscopy every hour for 72 hours. Worms were marked as green or non-green to indicate molting or non-molting, respectively. Worms that crawled off the side of the plate or burst were censored.

### Germline development

Plated animals, treated for 24 hours on RNAi or drug since hatching, were imaged at 20x magnification (Zeiss Axio Imager), and individual germ cells were counted with the Cell Counter plugin on Fiji software [76].

### Pharyngeal pumping analysis

Plated animals, treated for the indicated time on drug or RNAi since hatching, were imaged via the Movie Recorder at 8ms exposure using the ZEN 2 software at 10x magnification (Zeiss Axio Imager). Animals with zero pumping were excluded.

### ATP, ADP, AMP measurements

1000 or 500 plated animals, treated for 24 or 48 hours on drug or RNAi since hatching respectively, were washed 3 times in M9 buffer (keeping ~100μl of supernatant at final wash), snap frozen in a dry ice/ethanol bath, and placed at -80°C until use. Frozen pellets were boiled for 15 minutes and spun down at 14,800g at 4°C. The supernatant was then diluted in dH_2_O (1/10) (Adapted from[77]). Samples were tested for protein content via Bradford analysis (Amresco M173-KIT), and ATP was assessed via the ENLITEN^®^ ATP Assay System (Promega).

To determine relative levels of ATP/ADP/AMP, we followed the same method as above, but did not dilute the supernatant. Protein supernatant was directly assayed via the ATP/ADP/AMP Assay Kit (University at Buffalo, Cat. # A-125) to determine total ATP/ADP/AMP in each sample; these values were then directly compared to determine relative ratios.

### Inductively-coupled plasma atomic emission spectroscopy analysis

8,000–10,000 (L4 stage) or 20,000–25,000 (L2 stage) animals, treated for the listed time on the listed RNAi clone, were collected into microcentrifuge tubes (tubes weighed beforehand) using isotonic buffer (150mM Choline Chloride, 1mM HEPES, pH 7.4 with NaOH, filter sterilized). Worms were washed 3 times over 30 minutes (pelleting at 1,000g/30s each time) to clear gut content and then finally pelleted at 12,000g/2min. Worm pellets were then dried at 60°C for 48 hours using a heat block. Worm pellets were weighed after drying, and ICP analysis of the samples was conducted by Dr. David Kililea, Children's Hospital Oakland Research Institute. Before ICP analysis, dried pellets were acid digested with Omnitrace 70% HNO_3_ at 60°C overnight. Samples were diluted with Omnitrace water for a final concentration of 5% HNO_3_. Derived metal content was normalized to dried worm pellet weights. Each animal is compared back to 24hr Control RNAi treated animals. 48hr Control RNAi animals are given as a reference for what the metal content of a chronologically matched animal would be; albeit animals that are L4-YA stage and thus 2–3 larger with higher food intake.

### *Streptomyces* co-culture

*Streptomyces Alboniger* (ATCC 12461), *Griseus* (ATCC 23345), or *Griseolus* (ATCC 3325) were grown at 26°C, shaking, in Tryptone-Yeast Extract Broth (5g Tryptone, 3g Yeast Extract in 1L dH_2_O, pH 7; taken from ATCC^®^ Medium 1877: ISP Medium 1) for 5 days before plating unless otherwise noted. Strains were plated on Yeast Malt Agar plates (HiMedia Laboratories, M424), and mixed 1 part to 3 parts 25x HT115 when used with worms. For the egg laying comparisons, 100ul *Saccharomyces cerevisiae* was also added to induce competition; to compare total number of eggs, worms were mounted at ~52hrs after dropping to food source, and imaged at 20x magnification with DIC (Zeiss Axio Imager). For testing dead HT115, 75ml/L of 2.5% Streptomycin was added to 25x HT115 and the mix was rocked for 24hrs at room temperature. This mixture was then used in place of the 25x HT115 above.

### Survival assay

For survival in the arrested state, worms were dropped on the listed RNAi and counted each day (for the majority) for survival. Survival was assessed by touch response to prodding with a platinum wire. The Control RNAi wild type control strain used in this experiment was moved each day starting at adult day 1 as necessary until reproduction ceased.

For tissue-specific lifespan analysis, worms were grown on Control RNAi until L4/young adult age, and then transferred to the listed RNAi plates treated with 50μM FUdR. Survival was assessed every other day as above.

For all assays, animals were only censored (bursting, vulval protrusion, etc.) after the first counted death.

### Worm imaging

Worm morphological comparisons were imaged at 20x magnification with DIC filter (Zeiss Axio Imager). Worm length comparisons were made in ImageJ using the segmented line tool down the midline of each animal from head to tail.

For GFP and RFP reporter strains, worms were mounted in M9 with 10mM Sodium Azide, and imaged at 40x magnification with DIC and GFP/RFP filters (Zeiss Axio Imager). Fluorescence is measured via corrected total cell fluorescence (CTCF) via ImageJ and Microsoft Excel. CTCF = Integrated Density–(Area of selected cell X Mean fluorescence of background readings).

For imaging of heat-induced GFP expression via strain CL2070, plated animals were maintained at 20°C and fed RNAi since hatching. After 24hrs, one set of worms was shifted to 36°C for 3hr, while the other was mounted (as above) for the baseline 0hr time point. The baseline plate was also checked after 3 hours at room temperature as a control for any room temperature-induced GFP expression. Worms were imaged at 20x magnification with bright field and GFP filter (Zeiss Axio Imager).

### Statistical analyses

Thermotolerance, oxidative stress, and heavy metal stress were all compared using Fisher's Exact Test using the statistical software R [78]; specifically, the bars in each graph represent a unique set of biological replicates (2–6 independent biological replicates, see S1 Table) relative to its own independent control cohort (and the significance level relative to this control is indicated by the # of stars above each bar); this test is employed as we are comparing the categorical variables of Alive vs Dead, and data is presented as changes in survival. Comparison of all RNAi clones and CHX for protein synthesis rates under the *mlt-10p*::*GFP* promoter was performed using one-way ANOVA. Lifespan curves were compared and analyzed via Log-Rank using JMP Pro 12. qPCR, worm fluorescence, metal content, ATP/ADP/AMP levels, and pharyngeal pumping comparisons were made with Student's t test using Microsoft Excel. When comparing groups of three or more, Bonferroni multiple comparison post-correction was employed on Fisher's test, ANOVA, and t tests.

## Supporting information

S1 Fig Protein synthesis inhibition promotes a sustained developmental arrest state. *A*. Schematic placement of RPS-11 (green), EGL-45/EIF3A (red), IFG-1/EIF4G (light blue), and cycloheximide (purple) in ribosome biogenesis and processivity *B*-*C*. As compared to control RNAi treated animals (blue), protein synthesis inhibition by *egl-45* (red) or *rps-11* (green) RNAi impairs GFP biosynthesis in response to heat shock in animals expressing *hsp-16*.*2p*::*GFP* (*B*) (N = 14–17) or in response to reporter of developmental molting by *mlt-10p*::*GFP* (*C*) (N = 40–48 from 2 biological replicates). *hsp-16*.*2p*::*GFP* worms are 24hrs on RNAi at time of heat shock (the 0hr); *mtl-10p*::*GFP* worms are the same age as the listed hour post-feeding. Both fluorophores are measured via corrected total cell fluorescence (CTCF). *D*. *egl-45* or *rps-11* RNAi results in a sustained, greater than 10 days, developmental arrest at the L2 larval stage. *E*. Decreasing the total percentage of RNAi in the food (via mixing with Control RNAi) results in a dose-dependent response for developmental arrest. *F*. DIC comparisons of wild type, *egl-45* and *rps-11*-arrested animals, *daf-2(e1368)* dauers, and arrested starved L1 larvae grown at 25C (scale bar is 100um). The gross developmental size of *egl-45* and *rps-11* RNAi arrested animals are between dauers and starved L1 larvae. *G*. Worms with reduced protein synthesis grown at 20C or 25C for 24hrs are smaller than control RNAi-fed animals. * p< 0.025, ** p<0.005, *** p<0.0005, **** p<0.00005 (*B*, *G*: Student's t test); ** p<0.0017, **** p<0.000017 (*C*: One-way ANOVA). See also S1 Table.
(PDF)

S2 Fig Organismal stress resistance is a specific response to protein synthesis inhibition-induced L2 arrest. *A*-*F*. Absolute survival of protein synthesis inhibition-induced arrested L2 larvae by RNAi to *egl-45* (red) or *rps-11* (green) as compared to control RNAi (blue) when exposed to oxidative (*A*,*C*,*D*) or thermal (*B*,*E*,*F*) stress following 24 (*A*,*B*), 48 (*C*,*E*), or 240 (*D*,*F*) hours of arrest (N = 23–423 from 2–3 biological replicates). *G*-*H*. Flow chart (*G*) of RNAi clones screened that induce larval stage 2 arrest and the timeline of maintained arrest (*H*) (N = 15–35 from 3 biological replicates). *I*. *acn-1* (brown), *let-767* (purple), and *pan-1* (yellow) RNAi do not reduce protein synthesis to the same degree as *egl-45* or *rps-11* RNAi as compared via *mlt-10p*::*GFP* analysis (N = 40–48 from 2 biological replicates). *J*-*K*. L2 arrest induced without protein synthesis inhibition through *acn-1*, *let-767*, or *pan-1* RNAi does not result in the same stress resistance phenotypes (N = 151–312 from 2–3 biological replicates). *L*. Arrested L2 *ifg-1* mutants (light blue) have oxidative stress resistance (N = 82–388 from 2 biological replicates). *M*. Survival of *acn-1*, *let-767*, and *pan-1* dropped as synchronized L1s onto RNAi (N = 101–132 from 4 biological replicates) (*acn-1* vs *egl-45* or *rps-11* p<0.0001, Log-Rank test). * p<0.0083 (*I*: One-way ANOVA); * p< 0.01667, ** p<0.0033, *** p<0.00033, **** p<0.000033, (*J*-*K*, Fisher's exact test), * p<0.05, ** p<0.01, **** p<0.0001 (*L*, Fisher's exact test).
(PDF)

S3 Fig Organismal stress resistance is independent of the DAF-16 dauer pathway. *A-C*. *daf-16(mgDf47)* mutant animals still arrest under reduced protein synthesis (*A*) (N = 22–32 from 2 biological replicates) and still have oxidative (*B*) and thermal (*C*) stress resistance (N = 40–397 from 2–3 biological replicates). *D*. Arrest from reduced protein synthesis does not protect against 1% SDS, and the protein synthesis arrest at L2 occurs instead of dauer diapause as—*daf-2(e1368)* animals become susceptible to SDS rather than develop into SDS-resistant dauers. * p< 0.025, ** p<0.005, *** p<0.0005, **** p<0.00005 (*B-C*: Fisher's exact test). *** p<0.0001 (Q, Two-way ANOVA). See also S1 Table.
(PDF)

S4 Fig Tissue-specific protein synthesis inhibition responses. *A*. Model of *C*. *elegans* tissues. *B*-*P*. As compared to hypodermal specific RNAi (as shown in Fig 2) and relative to control RNAi (*B*, *E*, *H*, *K*, *I*), intestinal-specific (*E*-*G*, *L*, *M*-*N*) and germline-specific (*H*-*J*, *K*, *O*-*P*) RNAi targeting *egl-45* or *rps-11* have attenuated or undetectable responses to protein synthesis inhibition (arrest N = 225–311, pumping N = 22–26, oxidative/thermal N = 79–364 from 2–3 biological replicates). *Q*. RNAi of *egl-45* (red, p<0.001, Log-rank test) or *rps-11* (green, p<0.01, Log-rank test) only in the hypodermis (left) in post-developmental wild type animals is sufficient to induce lifespan extension as compared to control RNAi treated animals (blue); this is compared to wild type (right) increases in lifespan under RNAi of *egl-45* (red, p<0.0001, Log-rank test) or *rps-11* (green, p<0.05, Log-rank test) (N = 50–221 from 2 biological replicates). *R*-*U*. An *rps-11p*::*gfp* (*R*,*S*) and *egl-45p*::*mCherry* (*T*,*U*) reporter is detectable in multiple tissues at 24 hours (*R*,*T*) and 48 hours (*S*,*U*) of development. **** p<0.0001 (*K*-*L*, One-way ANOVA); * p< 0.025, ** p<0.005, *** p<0.0005, **** p<0.00005 (*M*-*P*, Fisher's exact test). See also S1 Table.
(PDF)

S5 Fig Transcriptional profiling of tissue general and tissue specific protein synthesis inhibition responses. *A*-*E*. Relative to tissue general RNAi (solid), hypodermal specific (Hyp) RNAi induces similar transcriptional responses to reduced expression of *egl-45* (*A*, *C*, *G*, *H*) or *rps-11* (*B*, *D*, *E*, *F*, *I*, *J*), while germline specific (Ger) and intestinal specific (Int) RNAi responses are attenuated (3 biological replicates). *K*. Post-developmental RNAi of *egl-45* (red) or *rps-11* (green) is sufficient to induce resistance to toxic levels of cadmium (5mM) (N = 126–198). *L*-*M*. 24-hour hypodermal reduction of protein synthesis is sufficient to provide similar resistance as whole-body (*L*), and whole-body resistance is largely lost in *mtl-1* and *mtl-2* double mutants (*M*) (N = 97–257 from 2 biological replicates). *N*. 48 and 120hr hypodermal reduction of protein synthesis increases *mtl-1* expression further (3 biological replicates). * p<0.05, ** p<0.01, *** p<0.001 (*A*-*J*, *N*-*P*, *N*: Student's t test); * p< 0.025, ** p<0.005, *** p<0.0005, **** p<0.00005 (*K*-*M*: Fisher's exact test). See also S1 Table.
(PDF)

S6 Fig Steady state levels of metals in response to protein synthesis inhibition. *A*-*C*. Quantification of ICP-AES analysis of steady state metal levels in protein synthesis inhibition treated wild type (*A*) or *mtl-1; mtl-2* mutant (*B*, *C*) animals. *D*. Comparison of *mtl-1*; *mtl-2* mutant animal total metal levels compared to wild type (4–7 biological replicates). * p< 0.025, ** p<0.005, *** p<0.0005, **** p<0.00005 (*A*-*D*: Student's t test). See also S1 Table.
(PDF)

S7 Fig Changes in pharyngeal pumping in response to protein synthesis inhibition. *A*. *ifg-1* mutant animals (light blue) have reduced pharyngeal pumping compared to wild type animals (N = 12–22 from 2 biological replicates). *B*. RNAi of *egl-45* (red) or *rps-11* (green) reduces pharyngeal pumping rate over 15 days of L2 arrest (no control is given for 240/360 hours as all control animals are post-developmental) (N = 7–14 from 2 biological replicates). *C*. The pharyngeal pumping decrease is not dependent on *daf-16* (N = 15–18 from 2 biological replicates). *D-E*. RNAi of *egl-45* (red) or *rps-11* (green) only in the germline does not decrease pumping to the same degree (*D*) and increases pumping when RNAi is restricted in the intestine (*E*) (N = 9–21 from 2 biological replicates). *F*. 24 hours of RNAi of *egl-45* (red) or *rps-11* (green) in post-developmental wild type animals is sufficient to reduce pharyngeal pumping as compared to control RNAi treated animals (blue) (N = 11–18 from 2 biological replicates). * p<0.05, ** p<0.01, *** p<0.001, **** p<0.0001 (*A*: Student's t test); * p<0.025, ** p<0.005, *** p<0.0005, **** p<0.00005 (*B-F*: One-way ANOVA). See also S1 Table.
(PDF)

S8 Fig Organismal responses to protein synthesis inhibition are mediated by AMPK signaling. *A*. Protein synthesis inhibition induces L2 arrest independent of AMPK signaling. *B*-*O*. *aak-2/AMPK* mutation (*B*, *D*, *F*, *I*, *J*, *K*, *M*, *N*) abolishes protein synthesis inhibition responses, that are restored by ectopic expression of AAK-2(aa1-321) (*uthIs248; CA-AMPK*) (*C*, *D*, *E*, *J*, *L*, *M*, *O*) (48hr timepoint shown; pumping N = 12–27 from 1–2 biological replicates, cadmium/oxidative/thermal N = 79–351 from 2–3 biological replicates). *F*-*I*. The increased expression of *mtl-1*, but not the reduced expression of *mtl-2*, in response to *egl-45* (red) or *rps-11* (green) RNAi, is dependent on *daf-16* (*G*), which is a known transcriptional regulator the *mtl-1* locus (*H*) (3 biological replicates). * p<0.05, ** p<0.01, *** p<0.001, **** p<0.0001 (*F*-*H*: Student's t test) * p<0.025, ** p<0.005, *** p<0.0005, **** p<0.00005 (*B*-*E*: One-way ANOVA; *I-O*: Fisher's exact test). See also S1 Table.
(PDF)

S9 Fig Organismal responses to protein synthesis inhibition may have evolved from interactions with microbes that generate protein synthesis inhibition xenobiotics. *A*. Table of *Streptomyces* species that produce xenobiotics that target eukaryotic protein synthesis. *B*. Exposure to *Streptomyces* species grown at stationary phase delays reproduction (N = 21–47 from 2 biological replicates). *C*. CHX strongly inhibits protein synthesis when assayed through the *mlt-10p*::*GFP* reporter (N = 16-48from 2 biological replicates). *D*. Relative to vehicle (water) treatment, animals exposed to cycloheximide (CHX) delay development in a dose-dependent manner (*D*). *E-G*. Development resumes from L2 (blue) to L4 (red), to adult (green), and reproductive adult (purple) when animals are moved from either control RNAi (*E*), *egl-45* RNAi (*F*) or *rps-11* RNAi (*G*) onto *rde-1* RNAi to impede RNA interference (N = 95–114 from 2 biological replicates). *H*-*M*. Developmentally arrested animals, exposed for 24hrs to CHX, are resistant to oxidative (*H*) and thermal (*I*) stress (N = 48–301 from 2 biological replicates), increase *mtl-1* expression (*J*) (3 biological replicates), have reduced ATP levels (*K*) (3 biological replicates), and have similar metal profiles as RNAi-mediated protein synthesis inhibition animals (*L-M*) (7 biological replicates). *N-Q*. Animals released and allowed to develop after 24hr exposure to CHX at hatching have a small but non-significant decrease in brood size (*N*) (N = 12–13), have a small increase in oxidative stress resistance (*O*) (N = 84–219 from 2 biological replicates), are delayed 16-20hrs in reproduction timing (*P*) (N = 241–418 from 2 biological replicates), and have normal pumping rates by physiological day 3 of adulthood (*Q*) (N = 15–16 from 2 biological replicates). *R-S*. The effects of *S*. *griseus* on pumping are dependent on living HT115 (*R*) (N = 21–30 from 2 biological replicates), and CHX has a dose-dependent effect on pumping rate (*S*) (N = 7–16). * p<0.0166 (*B*: Student's t test); * p<0.05, ** p<0.01, *** p<0.001, **** p<0.0001 (*C*, *J-M*, *P-R*: Student's t test; *H-I*, *O*: Fisher's exact test) * p<0.01, ** p<0.002, *** p<0.0002, **** p<0.00002 (*S*: One-way ANOVA). See also S1 Table.
(PDF)

S1 Table Summary of replicates. (XLSX)

S2 Table CHX recovery. Animals kept on 0.35mg/ml CHX for 24 or 48 hours are able to escape to become reproductive adults.
(XLSX)
