# Supplementary figures and images for: Hypodermal responses to protein synthesis inhibition induce systemic developmental arrest and AMPK-dependent survival in Caenorhabditis elegans
Source: PLoS Genet. 2018 Jul 18;14(7):e1007520. doi: 10.1371/journal.pgen.1007520 (PMC6066256; doi:10.1371/journal.pgen.1007520)

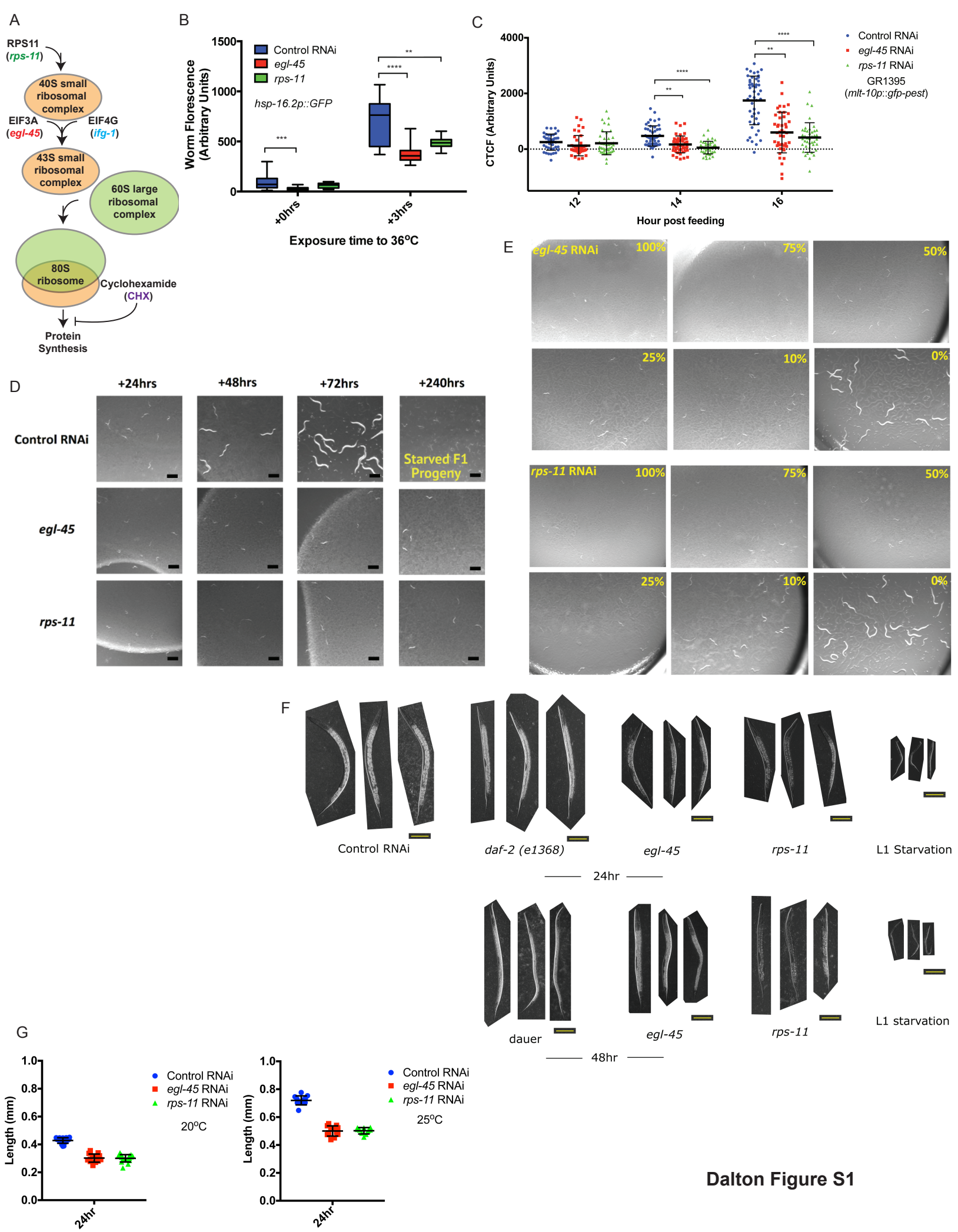

Dalton Figure S1

Supplement: S1 Fig — A. Schematic placement of RPS-11 (green), EGL-45/EIF3A (red), IFG-1/EIF4G (light blue), and cycloheximide (purple) in ribosome biogenesis and processivity B-C. As compared to control RNAi treated animals (blue), protein synthesis inhibition by egl-45 (red) or rps-11 (green) RNAi impairs GFP biosynthesis in response to heat shock in animals expressing hsp-16.2p::GFP (B) (N = 14–17) or in response to reporter of developmental molting by mlt-10p::GFP (C) (N = 40–48 from 2 biological replicates). hsp-16.2p::GFP worms are 24hrs on RNAi at time of heat shock (the 0hr); mtl-10p::GFP worms are the same age as the listed hour post-feeding. Both fluorophores are measured via corrected total cell fluorescence (CTCF). D. egl-45 or rps-11 RNAi results in a sustained, greater than 10 days, developmental arrest at the L2 larval stage. E. Decreasing the total percentage of RNAi in the food (via mixing with Control RNAi) results in a dose-dependent response for developmental arrest. F. DIC comparisons of wild type, egl-45 and rps-11-arrested animals, daf-2(e1368) dauers, and arrested starved L1 larvae grown at 25C (scale bar is 100um). The gross developmental size of egl-45 and rps-11 RNAi arrested animals are between dauers and starved L1 larvae. G. Worms with reduced protein synthesis grown at 20C or 25C for 24hrs are smaller than control RNAi-fed animals. * p< 0.025, ** p<0.005, *** p<0.0005, **** p<0.00005 (B, G: Student's t test); ** p<0.0017, **** p<0.000017 (C: One-way ANOVA). See also S1 Table. (PDF) [file pgen.1007520.s001.pdf]

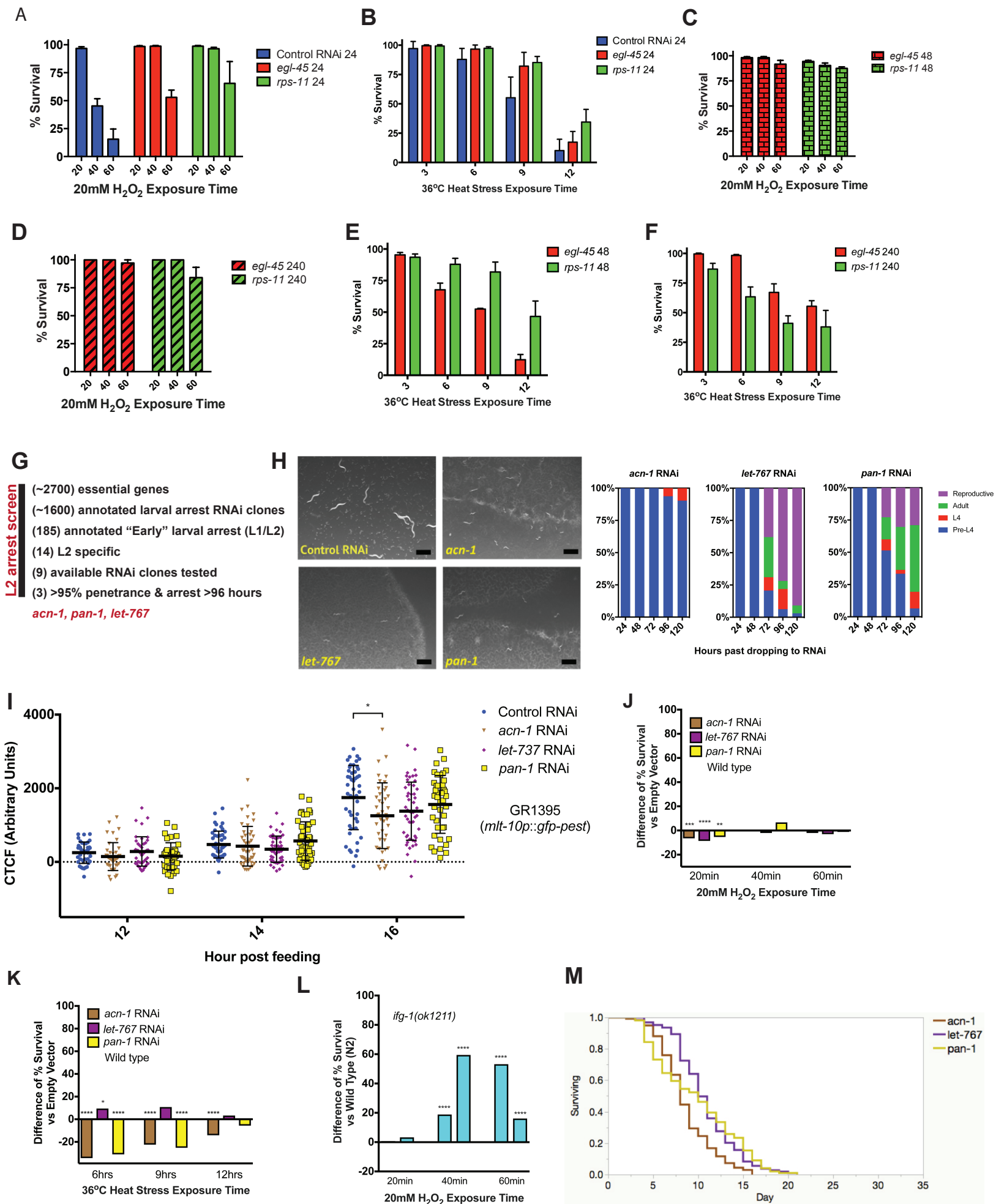

Supplement: S2 Fig — A-F. Absolute survival of protein synthesis inhibition-induced arrested L2 larvae by RNAi to egl-45 (red) or rps-11 (green) as compared to control RNAi (blue) when exposed to oxidative (A,C,D) or thermal (B,E,F) stress following 24 (A,B), 48 (C,E), or 240 (D,F) hours of arrest (N = 23–423 from 2–3 biological replicates). G-H. Flow chart (G) of RNAi clones screened that induce larval stage 2 arrest and the timeline of maintained arrest (H) (N = 15–35 from 3 biological replicates). I. acn-1 (brown), let-767 (purple), and pan-1 (yellow) RNAi do not reduce protein synthesis to the same degree as egl-45 or rps-11 RNAi as compared via mlt-10p::GFP analysis (N = 40–48 from 2 biological replicates). J-K. L2 arrest induced without protein synthesis inhibition through acn-1, let-767, or pan-1 RNAi does not result in the same stress resistance phenotypes (N = 151–312 from 2–3 biological replicates). L. Arrested L2 ifg-1 mutants (light blue) have oxidative stress resistance (N = 82–388 from 2 biological replicates). M. Survival of acn-1, let-767, and pan-1 dropped as synchronized L1s onto RNAi (N = 101–132 from 4 biological replicates) (acn-1 vs egl-45 or rps-11 p<0.0001, Log-Rank test). * p<0.0083 (I: One-way ANOVA); * p< 0.01667, ** p<0.0033, *** p<0.00033, **** p<0.000033, (J-K, Fisher's exact test), * p<0.05, ** p<0.01, **** p<0.0001 (L, Fisher's exact test). (PDF) [file pgen.1007520.s002.pdf]

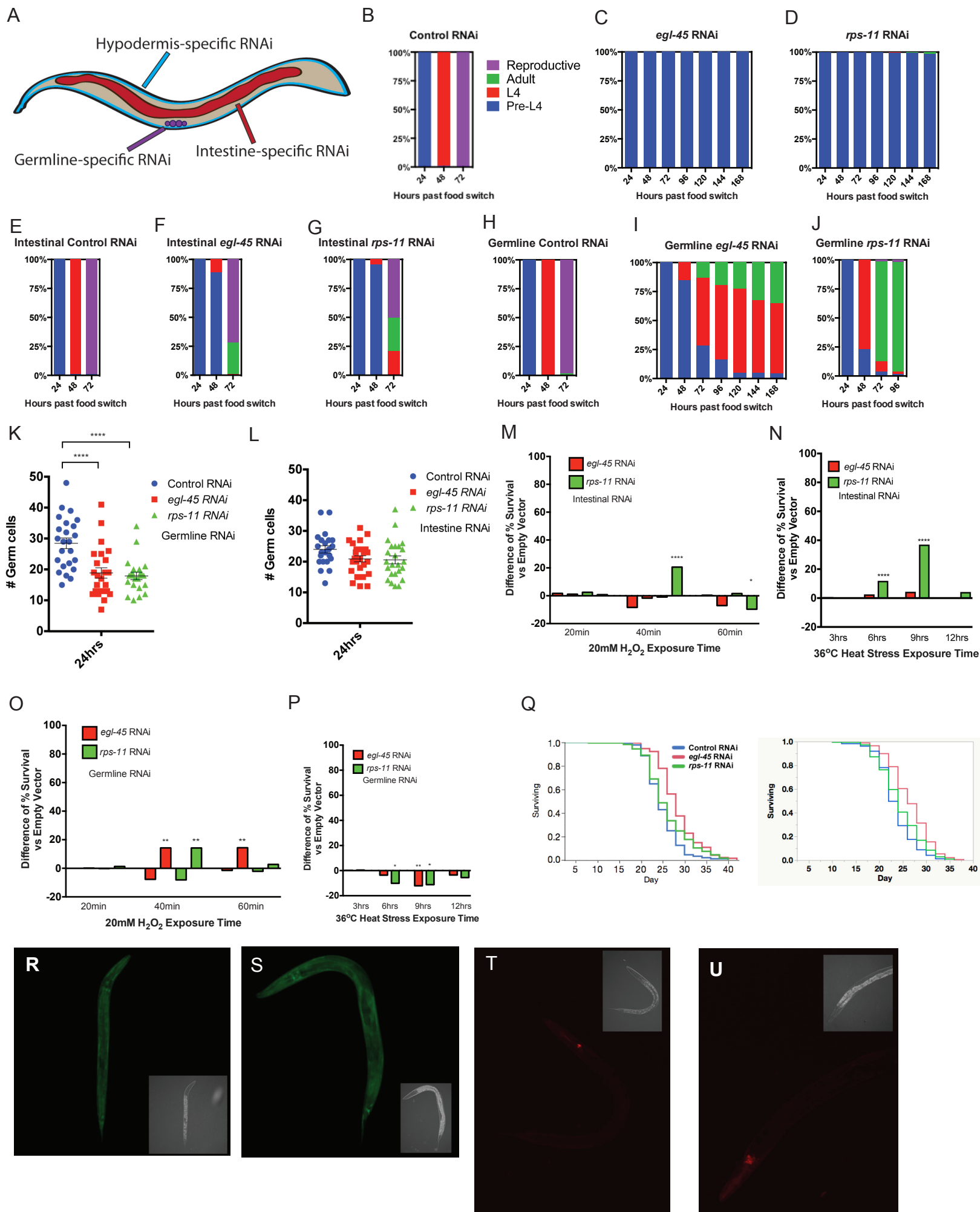

Dalton Figure S4

Supplement: S4 Fig — A. Model of C. elegans tissues. B-P. As compared to hypodermal specific RNAi (as shown in Fig 2) and relative to control RNAi (B, E, H, K, I), intestinal-specific (E-G, L, M-N) and germline-specific (H-J, K, O-P) RNAi targeting egl-45 or rps-11 have attenuated or undetectable responses to protein synthesis inhibition (arrest N = 225–311, pumping N = 22–26, oxidative/thermal N = 79–364 from 2–3 biological replicates). Q. RNAi of egl-45 (red, p<0.001, Log-rank test) or rps-11 (green, p<0.01, Log-rank test) only in the hypodermis (left) in post-developmental wild type animals is sufficient to induce lifespan extension as compared to control RNAi treated animals (blue); this is compared to wild type (right) increases in lifespan under RNAi of egl-45 (red, p<0.0001, Log-rank test) or rps-11 (green, p<0.05, Log-rank test) (N = 50–221 from 2 biological replicates). R-U. An rps-11p::gfp (R,S) and egl-45p::mCherry (T,U) reporter is detectable in multiple tissues at 24 hours (R,T) and 48 hours (S,U) of development. **** p<0.0001 (K-L, One-way ANOVA); * p< 0.025, ** p<0.005, *** p<0.0005, **** p<0.00005 (M-P, Fisher's exact test). See also S1 Table. (PDF) [file pgen.1007520.s004.pdf]

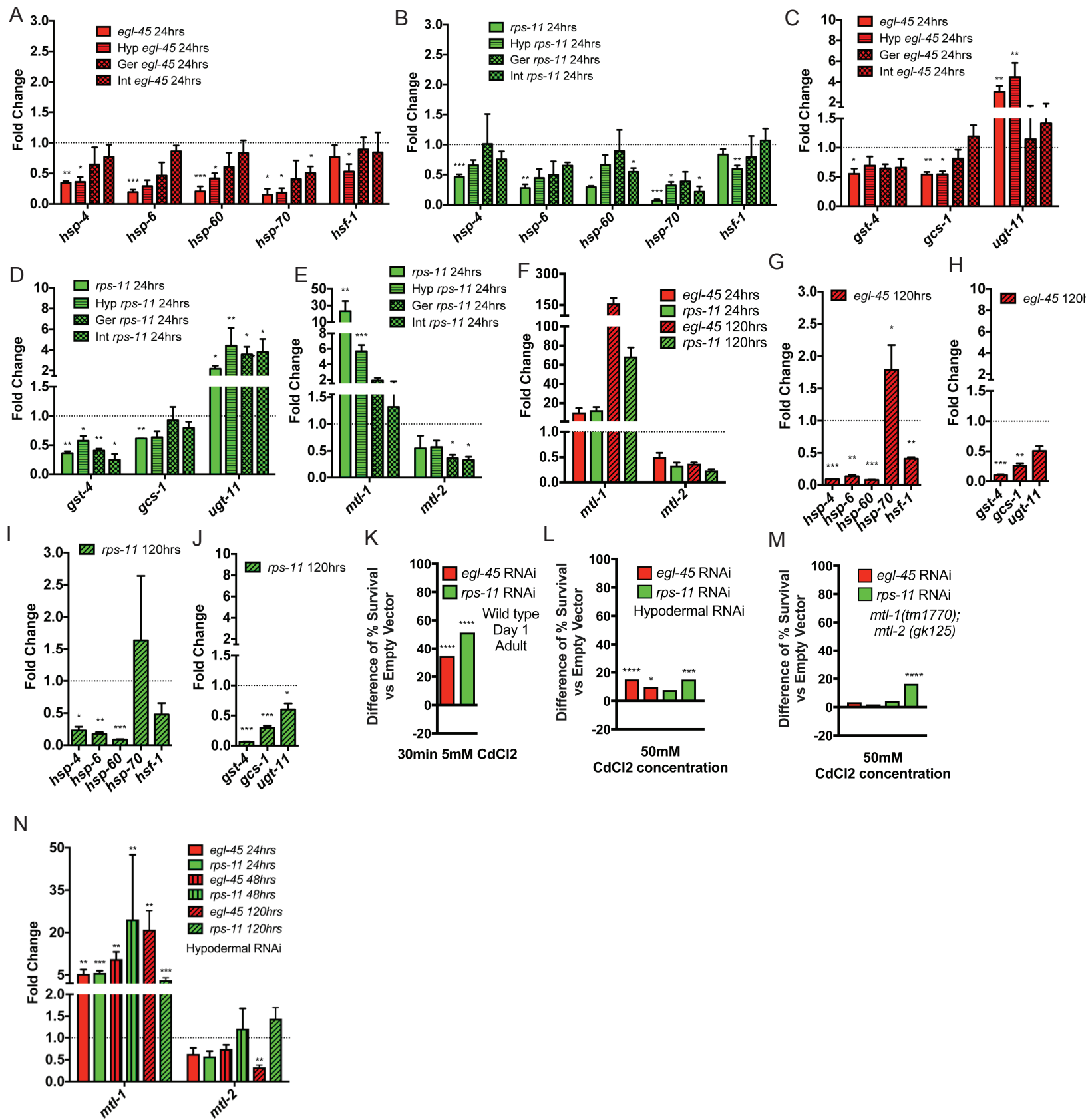

Dalton Figure S5

Supplement: S5 Fig — A-E. Relative to tissue general RNAi (solid), hypodermal specific (Hyp) RNAi induces similar transcriptional responses to reduced expression of egl-45 (A, C, G, H) or rps-11 (B, D, E, F, I, J), while germline specific (Ger) and intestinal specific (Int) RNAi responses are attenuated (3 biological replicates). K. Post-developmental RNAi of egl-45 (red) or rps-11 (green) is sufficient to induce resistance to toxic levels of cadmium (5mM) (N = 126–198). L-M. 24-hour hypodermal reduction of protein synthesis is sufficient to provide similar resistance as whole-body (L), and whole-body resistance is largely lost in mtl-1 and mtl-2 double mutants (M) (N = 97–257 from 2 biological replicates). N. 48 and 120hr hypodermal reduction of protein synthesis increases mtl-1 expression further (3 biological replicates). * p<0.05, ** p<0.01, *** p<0.001 (A-J, N-P, N: Student's t test); * p< 0.025, ** p<0.005, *** p<0.0005, **** p<0.00005 (K-M: Fisher's exact test). See also S1 Table. (PDF) [file pgen.1007520.s005.pdf]

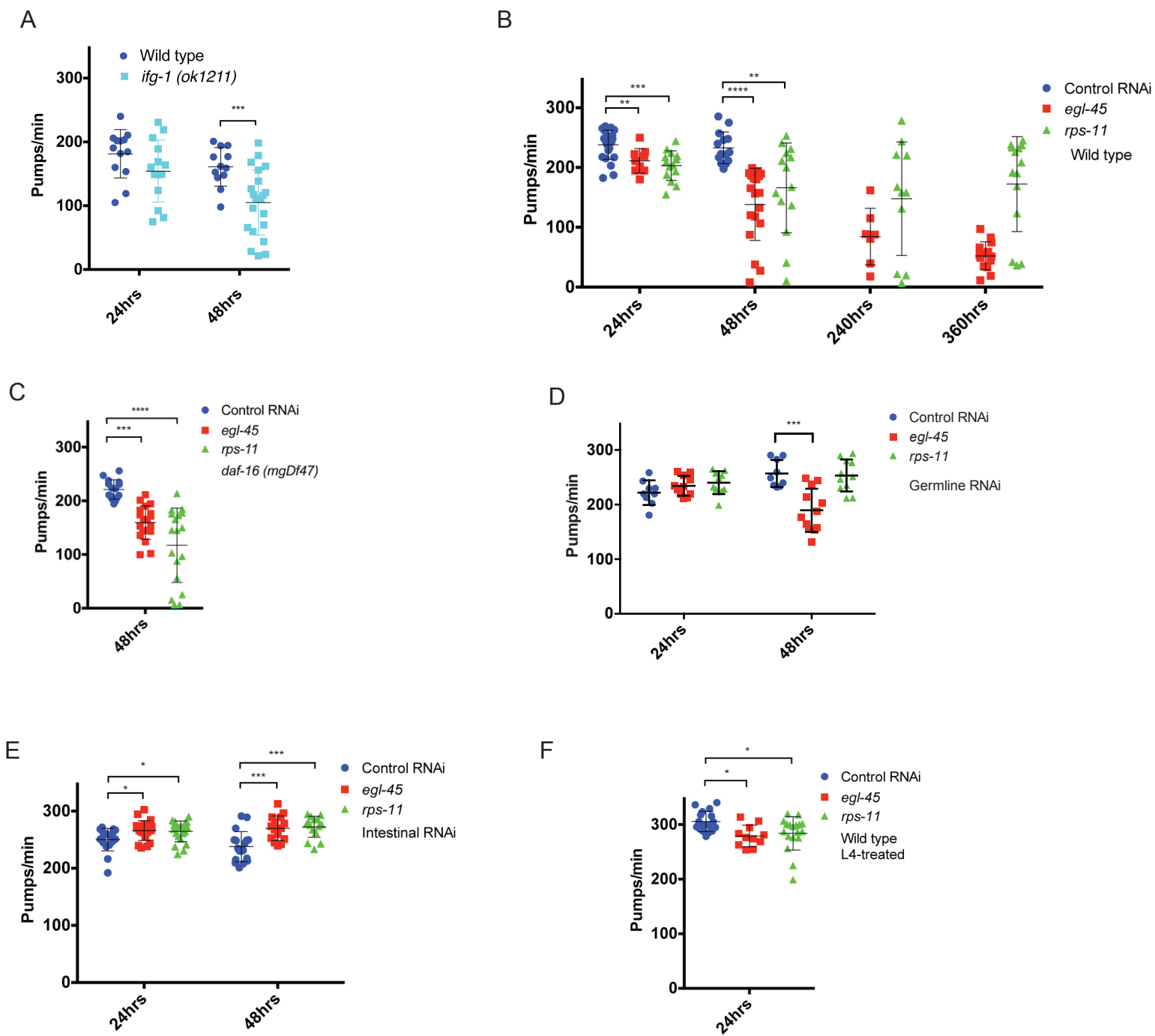

Supplement: S7 Fig — A. ifg-1 mutant animals (light blue) have reduced pharyngeal pumping compared to wild type animals (N = 12–22 from 2 biological replicates). B. RNAi of egl-45 (red) or rps-11 (green) reduces pharyngeal pumping rate over 15 days of L2 arrest (no control is given for 240/360 hours as all control animals are post-developmental) (N = 7–14 from 2 biological replicates). C. The pharyngeal pumping decrease is not dependent on daf-16 (N = 15–18 from 2 biological replicates). D-E. RNAi of egl-45 (red) or rps-11 (green) only in the germline does not decrease pumping to the same degree (D) and increases pumping when RNAi is restricted in the intestine (E) (N = 9–21 from 2 biological replicates). F. 24 hours of RNAi of egl-45 (red) or rps-11 (green) in post-developmental wild type animals is sufficient to reduce pharyngeal pumping as compared to control RNAi treated animals (blue) (N = 11–18 from 2 biological replicates). * p<0.05, ** p<0.01, *** p<0.001, **** p<0.0001 (A: Student's t test); * p<0.025, ** p<0.005, *** p<0.0005, **** p<0.00005 (B-F: One-way ANOVA). See also S1 Table. (PDF) [file pgen.1007520.s007.pdf]

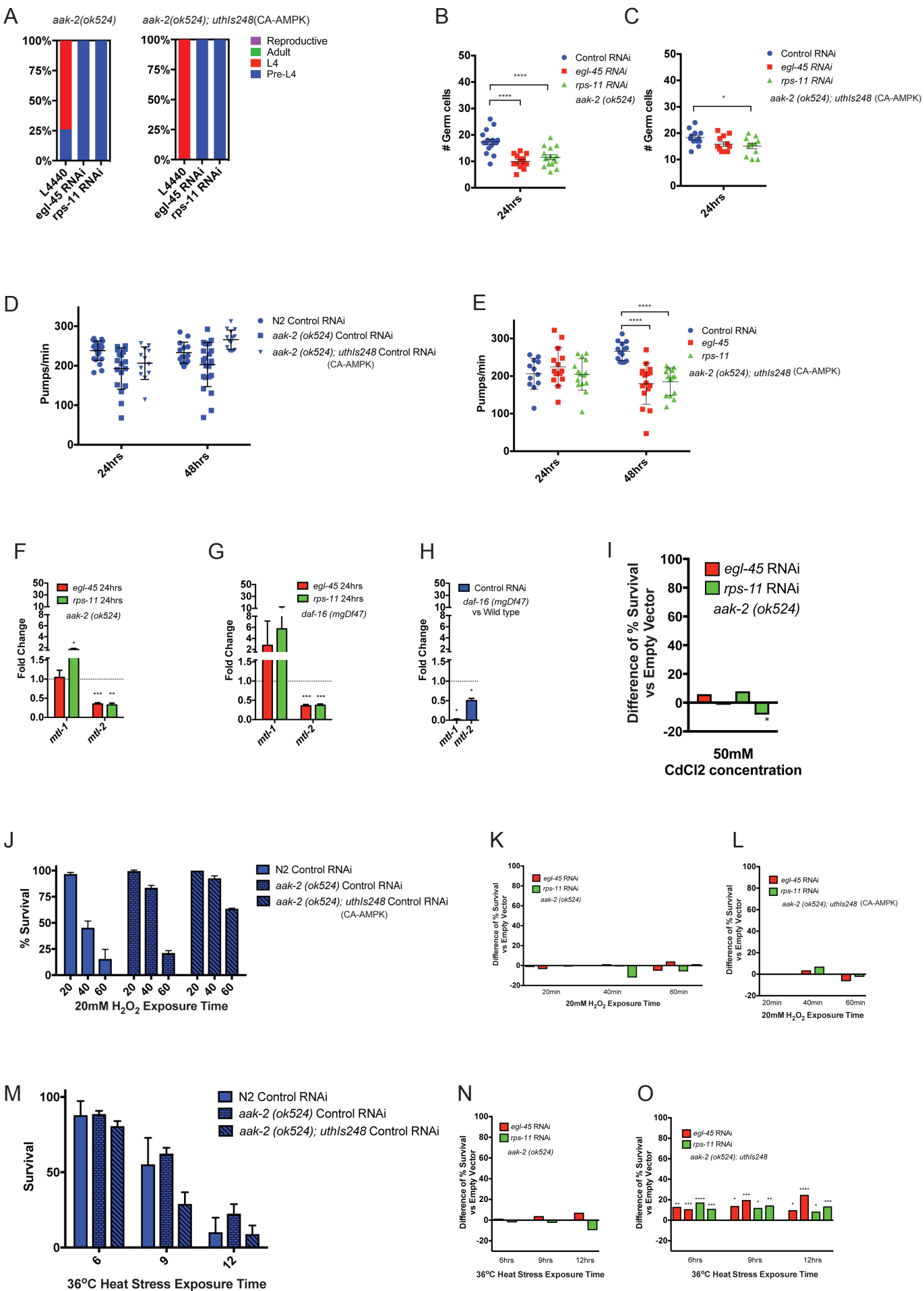

Dalton Figure S8

Supplement: S8 Fig — A. Protein synthesis inhibition induces L2 arrest independent of AMPK signaling. B-O. aak-2/AMPK mutation (B, D, F, I, J, K, M, N) abolishes protein synthesis inhibition responses, that are restored by ectopic expression of AAK-2(aa1-321) (uthIs248; CA-AMPK) (C, D, E, J, L, M, O) (48hr timepoint shown; pumping N = 12–27 from 1–2 biological replicates, cadmium/oxidative/thermal N = 79–351 from 2–3 biological replicates). F-I. The increased expression of mtl-1, but not the reduced expression of mtl-2, in response to egl-45 (red) or rps-11 (green) RNAi, is dependent on daf-16 (G), which is a known transcriptional regulator the mtl-1 locus (H) (3 biological replicates). * p<0.05, ** p<0.01, *** p<0.001, **** p<0.0001 (F-H: Student's t test) * p<0.025, ** p<0.005, *** p<0.0005, **** p<0.00005 (B-E: One-way ANOVA; I-O: Fisher's exact test). See also S1 Table. (PDF) [file pgen.1007520.s008.pdf]

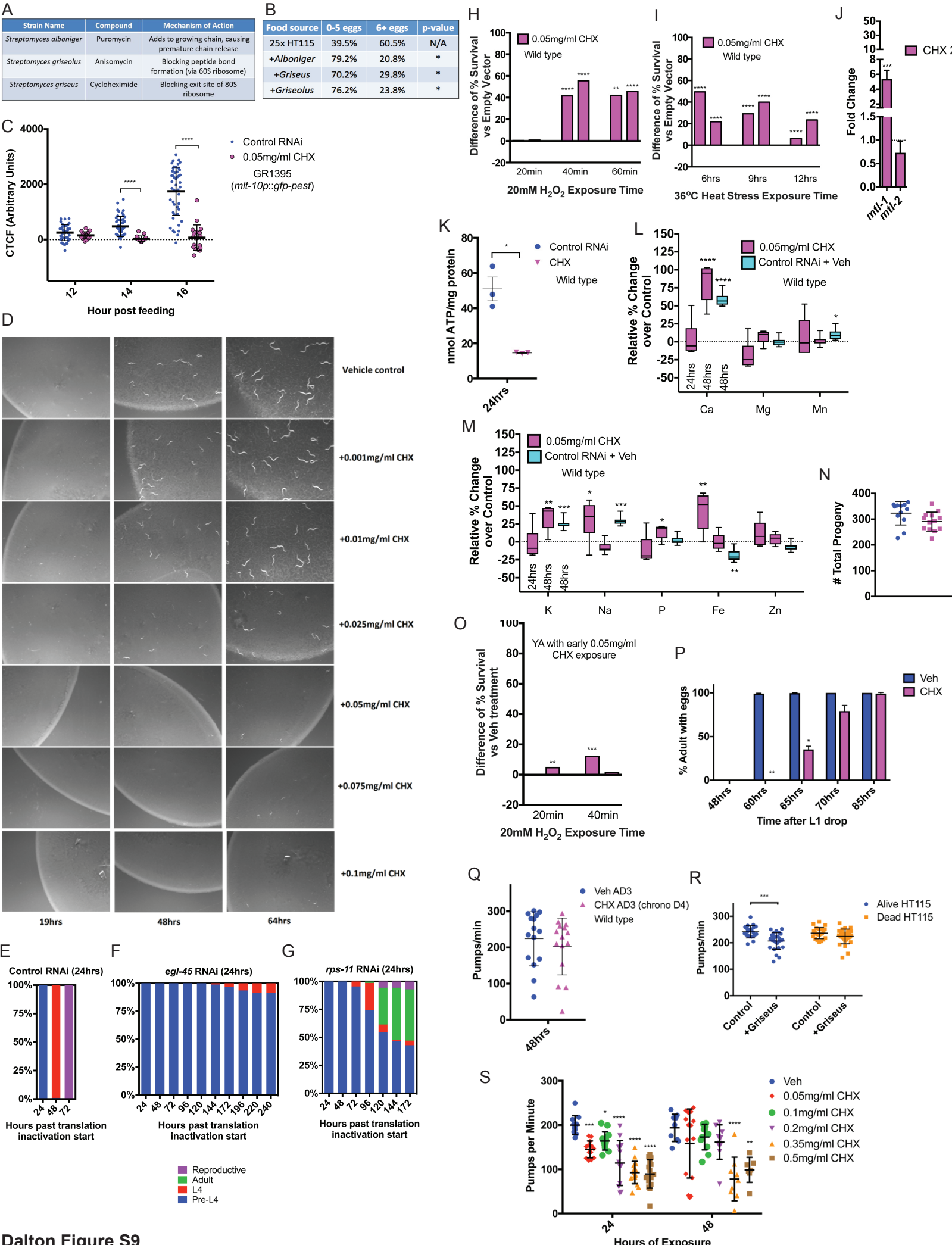

Dalton Figure S9

Supplement: S9 Fig — A. Table of Streptomyces species that produce xenobiotics that target eukaryotic protein synthesis. B. Exposure to Streptomyces species grown at stationary phase delays reproduction (N = 21–47 from 2 biological replicates). C. CHX strongly inhibits protein synthesis when assayed through the mlt-10p::GFP reporter (N = 16-48from 2 biological replicates). D. Relative to vehicle (water) treatment, animals exposed to cycloheximide (CHX) delay development in a dose-dependent manner (D). E-G. Development resumes from L2 (blue) to L4 (red), to adult (green), and reproductive adult (purple) when animals are moved from either control RNAi (E), egl-45 RNAi (F) or rps-11 RNAi (G) onto rde-1 RNAi to impede RNA interference (N = 95–114 from 2 biological replicates). H-M. Developmentally arrested animals, exposed for 24hrs to CHX, are resistant to oxidative (H) and thermal (I) stress (N = 48–301 from 2 biological replicates), increase mtl-1 expression (J) (3 biological replicates), have reduced ATP levels (K) (3 biological replicates), and have similar metal profiles as RNAi-mediated protein synthesis inhibition animals (L-M) (7 biological replicates). N-Q. Animals released and allowed to develop after 24hr exposure to CHX at hatching have a small but non-significant decrease in brood size (N) (N = 12–13), have a small increase in oxidative stress resistance (O) (N = 84–219 from 2 biological replicates), are delayed 16-20hrs in reproduction timing (P) (N = 241–418 from 2 biological replicates), and have normal pumping rates by physiological day 3 of adulthood (Q) (N = 15–16 from 2 biological replicates). R-S. The effects of S. griseus on pumping are dependent on living HT115 (R) (N = 21–30 from 2 biological replicates), and CHX has a dose-dependent effect on pumping rate (S) (N = 7–16). * p<0.0166 (B: Student's t test); * p<0.05, ** p<0.01, *** p<0.001, **** p<0.0001 (C, J-M, P-R: Student's t test; H-I, O: Fisher's exact test) * p<0.01, ** p<0.002, *** p<0.0002, **** p<0.00002 (S: [file pgen.1007520.s009.pdf]
