# Supplementary material for: Hypodermal responses to protein synthesis inhibition induce systemic developmental arrest and AMPK-dependent survival in Caenorhabditis elegans
Source: PLoS Genet. 2018 Jul 18;14(7):e1007520. doi: 10.1371/journal.pgen.1007520 (PMC6066256; doi:10.1371/journal.pgen.1007520)
Supplement: S3 Fig — A-C. daf-16(mgDf47) mutant animals still arrest under reduced protein synthesis (A) (N = 22–32 from 2 biological replicates) and still have oxidative (B) and thermal (C) stress resistance (N = 40–397 from 2–3 biological replicates). D. Arrest from reduced protein synthesis does not protect against 1% SDS, and the protein synthesis arrest at L2 occurs instead of dauer diapause as—daf-2(e1368) animals become susceptible to SDS rather than develop into SDS-resistant dauers. * p< 0.025, ** p<0.005, *** p<0.0005, **** p<0.00005 (B-C: Fisher's exact test). *** p<0.0001 (Q, Two-way ANOVA). See also S1 Table. (PDF) [file pgen.1007520.s003.pdf]

A B C

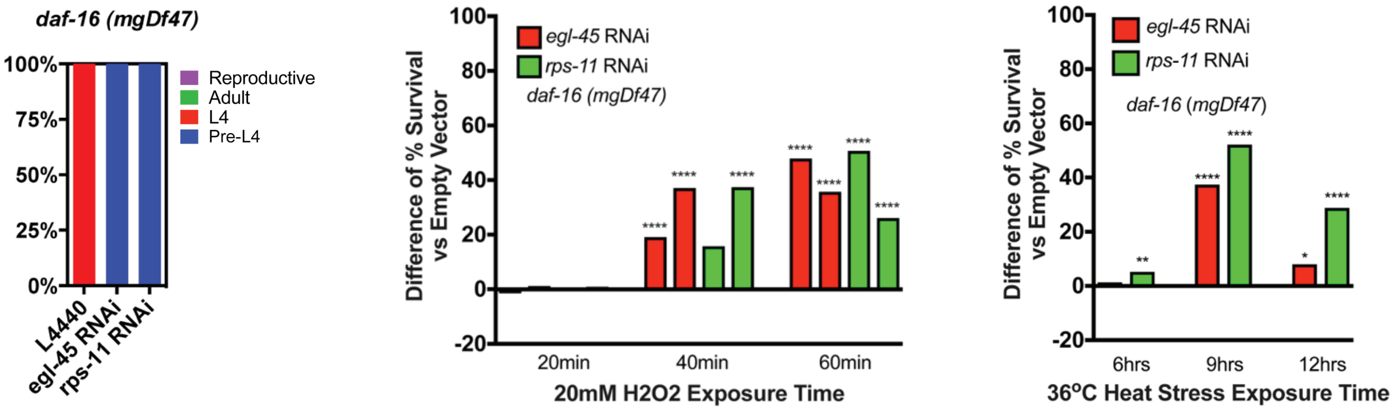

D

48hrs	30min 1% SDS					
**Genotype**	**RNAi/Drug**	**Alive**	**Dead**		**p-value**	**2-way ANOVA**
Wild type	Control RNAi	**0**	**140**	0%		
Wild type	*egl-45*	**0**	**34**	0%	>0.9999	ns
Wild type	*rps-11*	**0**	**71**	0%	>0.9999	ns
Wild type	0.05mg/ml CHX	**0**	**100**	0%	>0.9999	ns
daf-2 (e1368)	Control RNAi	**106**	**7**	94%		
daf-2 (e1368)	*egl-45*	**0**	**105**	0%	<0.0001	***
daf-2 (e1368)	*rps-11*	**0**	**150**	0%	<0.0001	***
daf-2 (e1368)	0.05mg/ml CHX	**36**	**118**	23%	<0.0001	***

Dalton Figure S3
