# Supplementary material for: Hypodermal responses to protein synthesis inhibition induce systemic developmental arrest and AMPK-dependent survival in Caenorhabditis elegans
Source: PLoS Genet. 2018 Jul 18;14(7):e1007520. doi: 10.1371/journal.pgen.1007520 (PMC6066256; doi:10.1371/journal.pgen.1007520)
Supplement: S6 Fig — A-C. Quantification of ICP-AES analysis of steady state metal levels in protein synthesis inhibition treated wild type (A) or mtl-1; mtl-2 mutant (B, C) animals. D. Comparison of mtl-1; mtl-2 mutant animal total metal levels compared to wild type (4–7 biological replicates). * p< 0.025, ** p<0.005, *** p<0.0005, **** p<0.00005 (A-D: Student's t test). See also S1 Table. (PDF) [file pgen.1007520.s006.pdf]

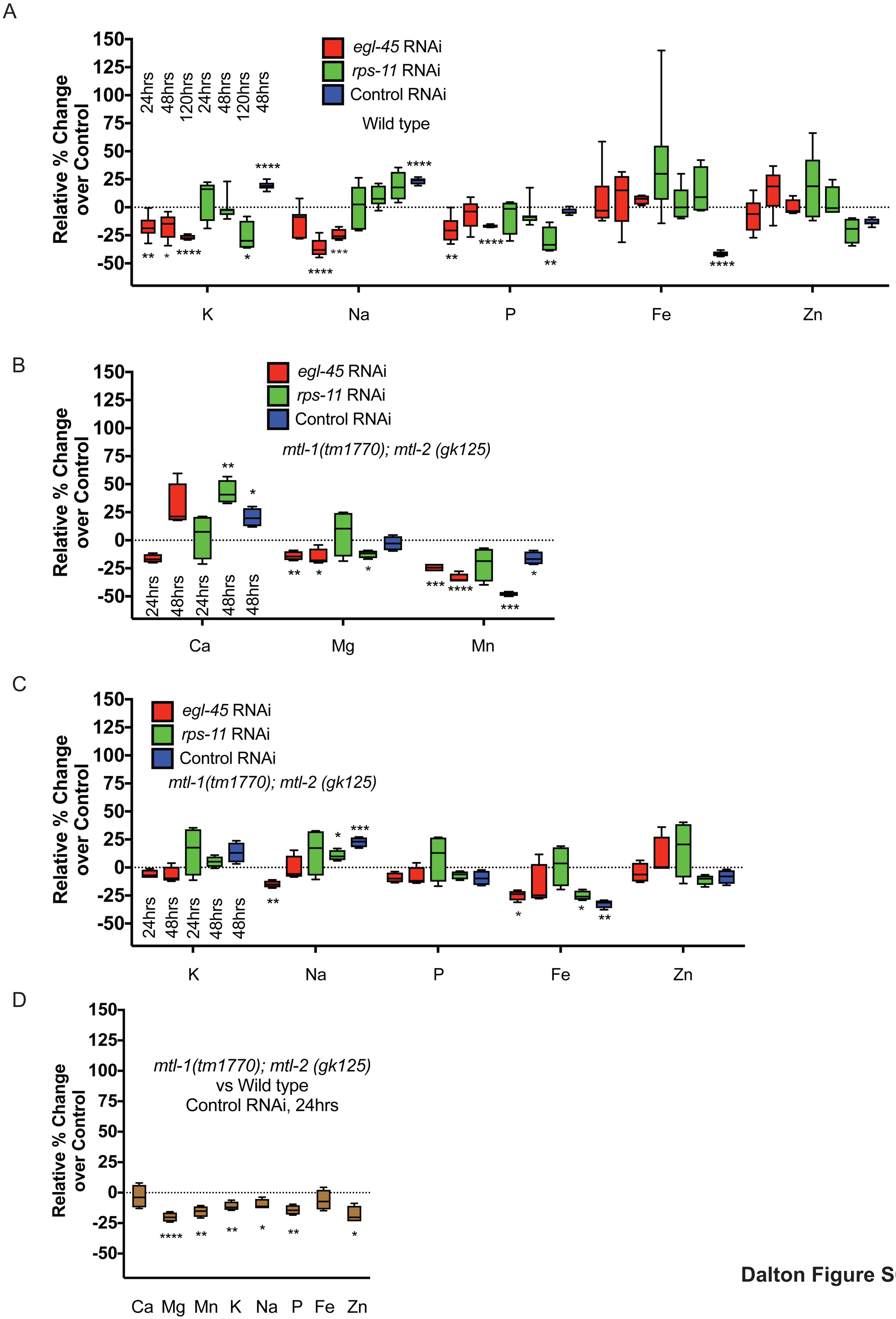

Dalton Figure S6
